# Mining telemonitored physiological data and patient-reported outcomes of congestive heart failure patients

**DOI:** 10.1371/journal.pone.0190323

**Published:** 2018-03-01

**Authors:** Miha Mlakar, Paolo Emilio Puddu, Maja Somrak, Silvio Bonfiglio, Mitja Luštrek, on behalf of the Chiron and HeartMan research projects

**Affiliations:** 1 Department of Intelligent Systems, Jožef Stefan Institute, Ljubljana, Slovenija; 2 Department of Cardiovascular, Respiratory, Nephrological, Anesthesiological and Geriatric Sciences, Sapienza University of Rome, Rome, Italy; 3 Fimi Barco, Saronno, Italy; Kurume University School of Medicine, JAPAN

## Abstract

This paper addresses patient-reported outcomes (PROs) and telemonitoring in congestive heart failure (CHF), both increasingly important topics. The interest in CHF trials is shifting from hard end-points such as hospitalization and mortality, to softer end-points such health-related quality of life. However, the relation of these softer end-points to objective parameters is not well studied. Telemonitoring is suitable for collecting both patient-reported outcomes and objective parameters. Most telemonitoring studies, however, do not take full advantage of the available sensor technology and intelligent data analysis. The Chiron clinical observational study was performed among 24 CHF patients (17 men and 7 women, age 62.9 ± 9.4 years, 15 NYHA class II and 9 class III, 10 of ishaemic, aetiology, 6 dilated, 2 valvular, and 6 of multiple aetiologies or cardiomyopathy) in Italy and UK. A large number of physiological and ambient parameters were collected by wearable and other devices, together with PROs describing how well the patients felt, over 1,086 days of observation. The resulting data were mined for relations between the objective parameters and the PROs. The objective parameters (humidity, ambient temperature, blood pressure, SpO2, and sweeting intensity) could predict the PROs with accuracies up to 86% and AUC up to 0.83, making this the first report providing evidence for ambient and physiological parameters to be objectively related to PROs in CHF patients. We also analyzed the relations in the predictive models, gaining some insights into what affects the feeling of health, which was also generally not attempted in previous investigations. The paper strongly points to the possibility of using PROs as primary end-points in future trials.

## Introduction

Congestive heart failure (CHF) is a chronic condition in which the heart cannot pump enough blood to meet the needs of organs and tissues for oxygen and nutrients. It can have a variety of causes, including damaged heart tissue (e.g., due to a heart attack), atherosclerosis, hypertension etc. CHF is the most frequent cause of hospitalization in people aged over 65, and costs the society around 100 billion USD per year [1]. Since it cannot be cured, successful management of CHF if critical for the health outcomes and quality of life of the patients, as well keeping healthcare expenses in check.

Telemonitoring appears a promising approach for CHF, with two recent systematic reviews showing a reduction in mortality and the number of hospitalizations [2, 3]. However, this success is not universal, since some large trials showed no benefit from telemonitoring [4]. This indicates that telemonitoring in CHF deserves further research. One direction of such research is to include patient-reported outcomes (PROs) such as symptoms or health-related quality of life, since the quality of life is an important goal independent of “hard” outcomes such as mortality and hospitalizations. Another direction is to improve the telemonitoring technology using wearable technology and intelligent computer methods, since most telemonitoring trials so far used relatively simple technology [5].

PROs are valued by patients, clinicians and policy-makers, and they inform therapeutic choices, disease management practices, reimbursement decisions, and health policy [6]. The European Society of Cardiology confirmed the importance of PROs in cardiovascular clinical research by suggesting they should be included in the evaluation of the efficacy of therapeutic interventions, provided the PROs are assessed scientifically and rigorously [7]. The call for reducing the burden of disease [8, 9], and not only prolonging the life but also improving its quality (by using the quality-adjusted life year as a metric [10]) is heard in many places. Unfortunately however, these emerging goals, with a few exceptions in specific areas of transplantation, have very little on which to rely [11, 12]. Telemonitoring appears to be a suitable solution to this problem, since it can easily collect PROs together with physiological data.

Wearable technology is currently improving rapidly both in consumer and medical areas. Unlike traditional telemonitoring, where single daily measurements of parameters such as heart rate and blood pressure are made, it can generate large amounts of data providing richer picture of the patient’s condition. It goes hand-in-hand with intelligent computer methods such as machine learning and data mining, which can interpret these data and support patients and physicians in disease management and treatment. However, given the relative simplicity of most past telemonitoring trials, the knowledge on how to interpret data from wearable devices is underdeveloped.

Tripoliti et al. [13] performed a comprehensive review of machine learning and data mining applied to all aspects of the management of CHF. Most of the reviewed work used clinical data, which significantly differs in type and sampling frequency from telemonitoring data. There were only two cases where telemonitoring data was used [14, 15], both of which predicted re-hospitalizations. Koulaouzidis et al. [14] built a naive Bayes classifier with blood pressure, heart rate and weight as features, yielding the area under the ROC curve (AUC) of 0.82. Kang et al. [15] built a J48 decision tree with PROs and other parameters as features, yielding the AUC of 0.59. Sudden weight gain in patients living with CHF is often an indication of retaining fluid, which increases the risk of kidney or cardiac failure. Fisher et al. [16] thus developed a latent variable autoregression model that tracks patient weight and blood pressure over time to predict weight values and provide early warning of fluid retention. Pecchia et al. [17] proposed a telemonitoring platform that can detect the presence and severity of CHF using a CART decision tree build on heart-rate variability features from an electrocardiogram (ECG) monitor. The AUC for the presence was 0.95 and for the severity 0.79. The practical utility of these results is limited, though, since most people consent to telemonitoring only after they are diagnosed with CHF.

This paper reports the result of the Chiron clinical observational study performed among CHF patients in Italy and UK, in whom a large number of physiological and ambient parameters were collected by wearable and other devices, together with PROs describing how well the patients felt, over 1,086 days of observation. The resulting data were preprocessed and mined for relations between the objective parameters and the PROs. The objective parameters could predict the PROs quite accurately, making this the first report providing evidence for ambient and physiological parameters to be objectively related to PROs in CHF patients. Predicting PROs was not attempted in any of the related work we found, and is probably more challenging than predicting “hard” outcomes such as hospitalizations. We also analyzed the relations in the predictive models, gaining some insights into what affects the feeling of health, which was also generally not attempted in related work.

## Materials and methods

### Observational study

The overall objective of the Chiron project (an Artemis funded European Project) was to develop a comprehensive framework for personalized health management comprising mobile, home and hospital services. A part of the Chiron framework intended for CHF patients was a decision-support system whose objective was to estimate the health risk of the patients [18]. However, since there was not enough knowledge on how to associate the values of the various measured parameters with the risk, an observational study was carried out in the project with the intention to generate such knowledge.

Before starting the study, the protocol was submitted to the ethics committes of Umberto I polyclinic for the Italian part of the study, and South East Scotland Research Ethics Committee for the UK part of the study. Ethical approval was obtained in each country, and the study carried out according to the national regulations. All the participants gave written informed consent.

In the observational study the CHF patients were classified into NYHA class II or III, both with coronary heart disease and non-ischemic aetiologies. They were all on optimal medical therapy according to their individual practicing cardiologists, which was stable for six weeks before recruitment (based on NICE Guidelines). All had sinus rhythm and were without major cardiac arrhythmias on standard ECG. All had reduced ejection fraction based on 2D echocardiography and, depending on individual aetiologies, either increased or dilated left ventricular volumes. There were no patients with plain respiratory insufficiency. The patients were able to use the telemonitoring devices, had no implanted devices and were not on a heart-transplant list.

**Fig 1:**
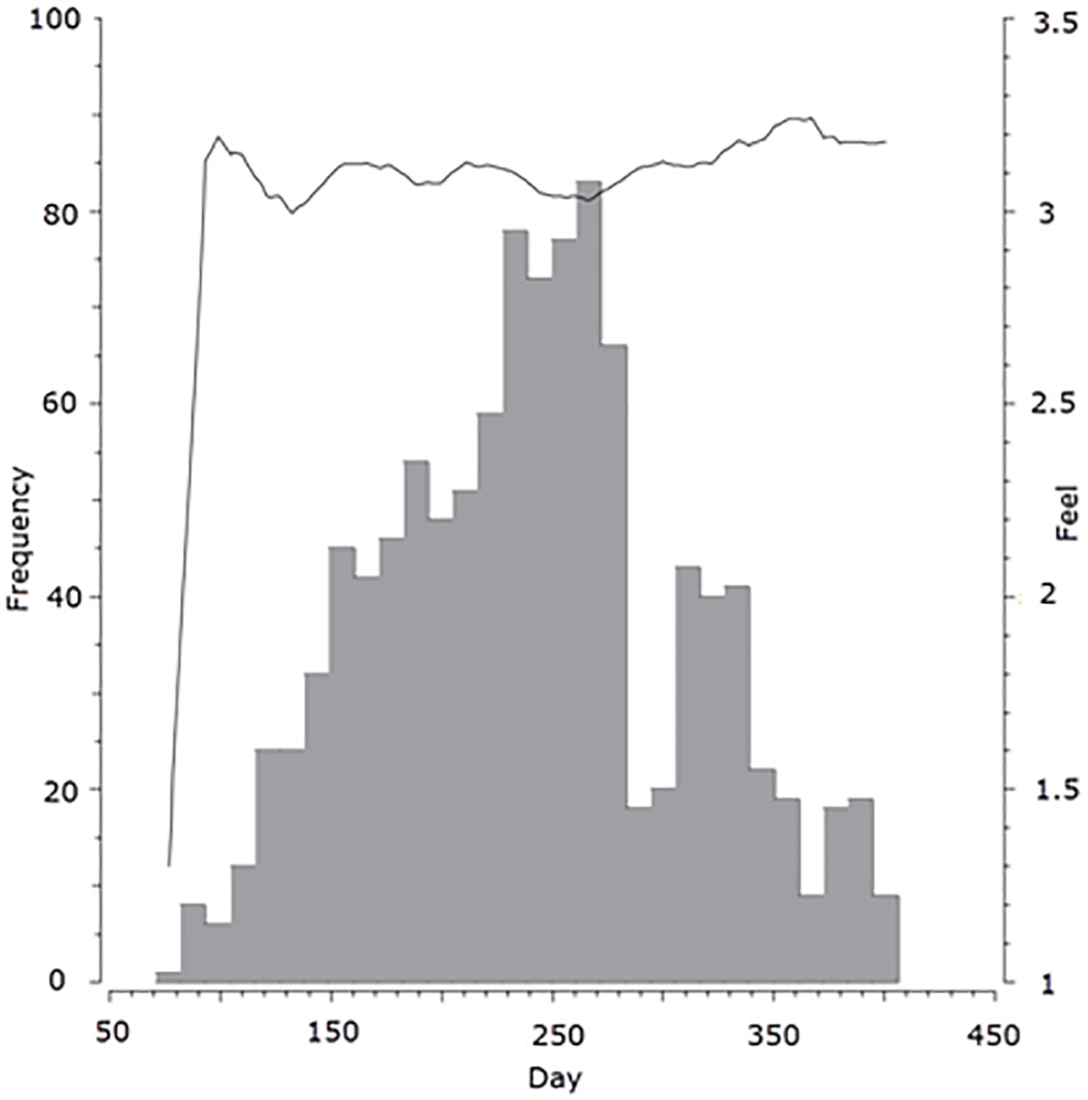
Number of observations obtained on each day of the study.

The Chiron patients were equipped with a wearable ECG, activity, body-temperature and sweat sensors. In addition, their blood pressure, blood oxygen saturation, weight, and ambient temperature and humidity were measured. The patients were instructed to perform daily measurements with the non-wearable devices, and use wearable devices for two hours in the morning and twice for one hour in the afternoon. The patients were also provided with a mobile application for reporting their overall feeling of health with respect to the previous day on a daily basis. They marked their overall feeling of health as one of the following options: feeling much worse than yesterday (1 in Fig 1 and elsewhere in the paper), feeling worse than yesterday (2), feeling the same as yesterday (3), feeling better than yesterday (4) and feeling much better than yesterday (5). The study also intended to gather data about hospital admissions and deaths, but no such events occurred during the study period (patients observations lasted from 1 to 84 days). Therefore, we decided to use the patients’ self-reported feeling of health (PROs) instead.

The whole study included 38 CHF patients: 19 from the United Kingdom and 19 from Italy. However, some of the data were incomplete, so only the data of 11 patients from the UK and 13 patients from Italy were included in the analysis. Among them were 17 men and 7 women, aged 62.9 ± 9.4 years, 15 belonging to NYHA class II and 9 to class III. 10 had ishaemic etiology, 6 dilated, 2 valvular, and 6 multiple etiologies or cardiomyopathy (younger patients).

**Fig 2:**
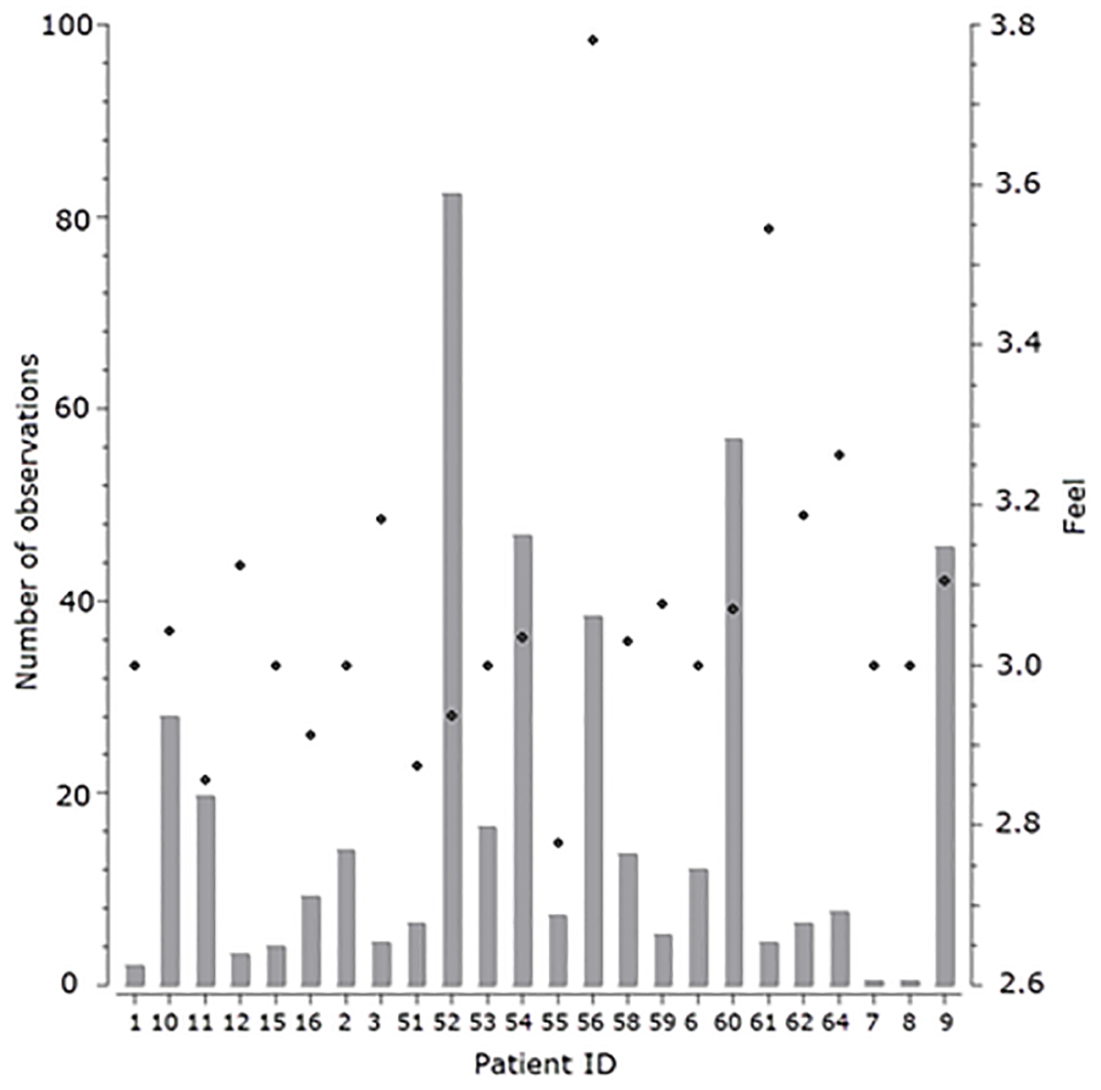
Number of observations obtained for each patient.

These 24 patients together provided a total of 1086 usable recording days. Fig 1 shows how many observations were obtained on which days of the study, and what the average feeling of health of the patients was. The bars represent the frequency of obtained observations during the study and the line represents the average feeling of health of the patients for those observations. Fig 2 presents the number of observations obtained for each patient, and their average feeling of health. Bars represent the number of obtained observations for each patient and point represents their average feeling of health. Most of the observations were obtained in the middle of the study, the average feeling of health was around 3 (patient feeling the same as yesterday), and the number of obtained observations varied greatly among the patients. All patients provided written informed consent for the participation in the study.

### Telemonitoring system and parameters

**Fig 3:**
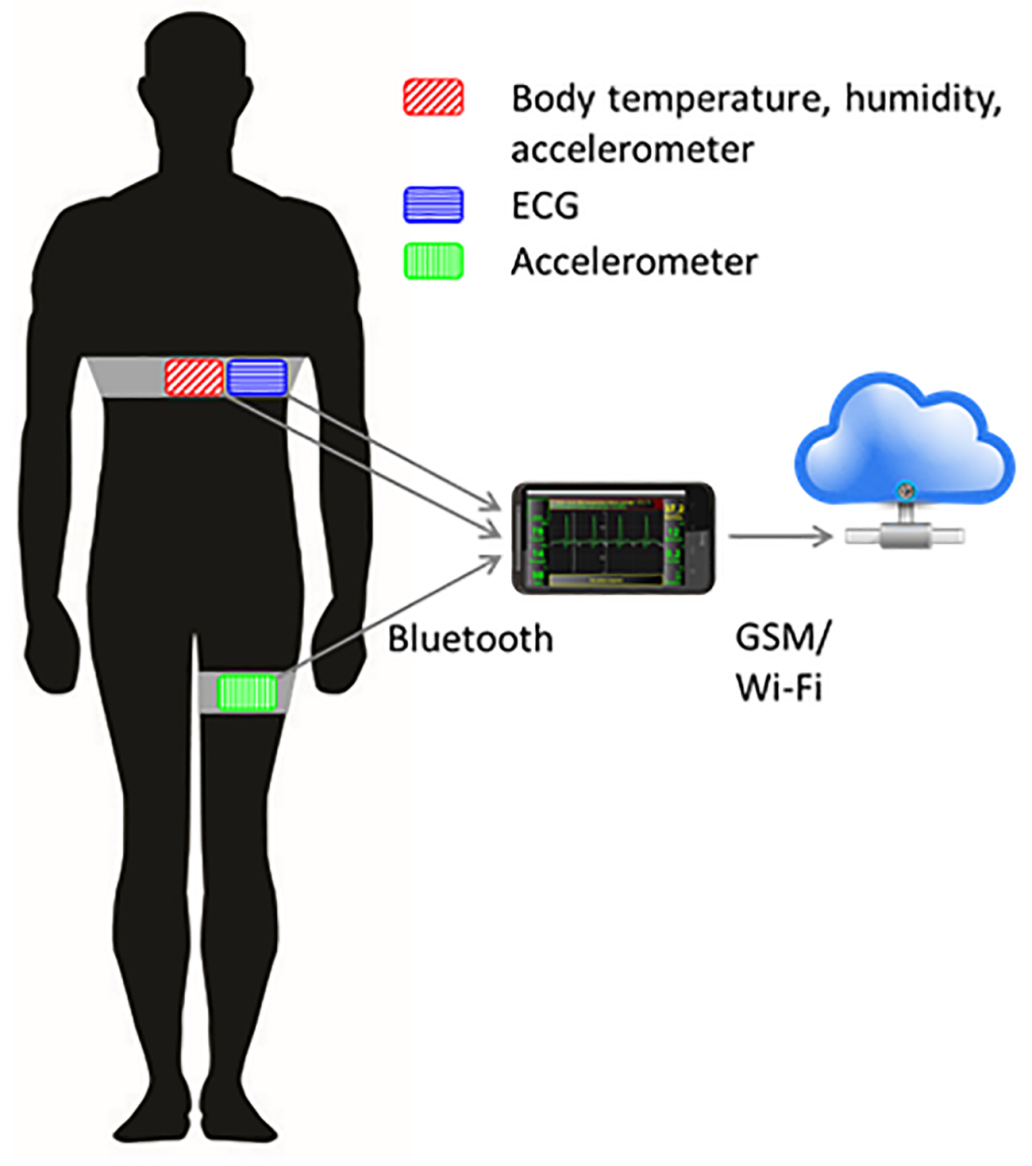
Chiron wearable sensor platform.

The main wearable device used was a chest strap with a single-lead ECG monitor, body-temperature and body-humidity (sweat) sensors, and an accelerometer. This accelerometer was complemented by another one on the thigh for activity monitoring. Fig 3 shows the wearable sensors worn by the patients in the study.

The wearable sensors transmitted their data to a smartphone. A smartphone application was additionally used to input daily measurements of blood pressure, peripheral capillary oxygen saturation (SpO_2_), weight, and ambient temperature and ambient humidity (HumAmbient).

The Falcon algorithm [12] was used to extract fiducial points from the ECG signal, enabling us to compute the heart rate as well as to describe each heart beat with additional parameters such as PR interval, QRS duration and QT interval.

Another algorithm [19] was used to recognize the patients’ physical activity based on the accelerometer signals. The recognized activity together with the accelerometer signals were further used to estimate the patient’s energy expenditure, which corresponds to the intensity of their activity.

Some parameters were monitored continuously, some were obtained on a daily basis and some were taken at the beginning of the study. The following continuously telemonitored parameters were considered for the analysis:

- Body humidity (sweating)
- Body temperature
- PR interval
- QRS duration
- T-wave amplitude
- QT interval
- RR interval (and thus heart rate)
- R-wave amplitude
- Duration of physical activity, which could be lying, sitting or moving
- The patient’s energy expenditure

The following parameters were measured on a daily basis:

- Systolic and diastolic blood pressure (SBP, DBP)
- SpO_2_
- Weight
- Ambient temperature
- Ambient humidity
- How the patient feels compared to the previous day

In addition to these measured parameters, for each patient, additional static parameters were taken at the hospital at the beginning of the study. These parameters included general patient information, current medical treatments, comorbidities and the results of blood analysis. Due to the small amount of patients participating in the study, the data mining algorithms could use the static parameters to model relations between the patient’s identity (determined by one or more of these parameters) and how they feel, which was not our objective. Accordingly, the static parameters were not considered for data mining.

### Feature construction

For every parameter that was measured continuously or multiple times per day we calculated the following features:

- Average value during the whole day
- Average value during each activity (during lying, sitting and moving)
- Standard deviation during the whole day
- Standard deviation during each activity (during lying, sitting and moving)

From the measured parameters described in the previous subsection, we further calculated additional features:

- Ratios between the durations of the three activities
- Ratio between the average skin and ambient temperature
- Ratio between the average skin and ambient humidity
- Rate pressure product [RPP = SBP * HR]; HR is the heart-rate measurement closest to the SBP measurement; if there is no HR measurement close to the SBP measurement, the daily average of heart-rate values
- Double product [DoP = (SBP + DBP)/2 * HR]; (SBP + DBP)/2 is the mean pressure product, HR is selected the same way as for the rate pressure product
- Ratio between systolic and diastolic blood pressure
- Differences between the average heart-rate values during all pairs of activities (lying–sitting, lying–moving, sitting–moving)
- Ratios between the average heart-rate values during all pairs of activities
- Fraction of time spent on each of the three physical activities during daily recordings
- Average ratio between the heart rate and energy expenditure, always using the closest measurements of both values

For all the features we also calculated:

- Change of the feature value in comparison with the previous day (or–if there is no measurement on the previous day–the change of daily value in comparison with the last daily value, if it is at most 3 days 'old')
- Personalized (normalized) feature value as the ratio between the daily value of the parameter and its average value for the patient over the whole study period.

### Feature selection

We constructed 244 features in total, which is a lot compared to the number of instances (recording days). Therefore we applied several feature-selection approaches and as a result obtained different subsets of features. These subsets of data were then used for building predictive models.

The first approach was to use two known automatic feature-selection techniques called RelieF [20] and Correlation-based Feature Subset Selection (CFS) [21]. In RelieF, for every feature, a relevance vector is calculated based on how nearby instances are classified (same class or not). All the features with their relevance greater than a predefined threshold are selected. The CFS approach does not evaluate each feature separately, but evaluates subsets of features on the basis of the hypothesis that good feature subsets contain features highly correlated with the class value, yet uncorrelated with each other. We tried both methods, but since CFS approach resulted in better models, we used only subsets obtained with the CFS approach. This subset is called “CFS_feature_selection”.

Another subset was chosen manually in a medically driven manner. The selection is based on general medical knowledge and literature, as well as previous experience from the Chiron project. This subset was called “Expert_selection”.

The features describing the patients’ activities had a lot of missing values, due to, for example, patients not wearing the sensors correctly. Consequently all the features related to specific activities (during lying, sitting and moving) had at least 60% of missing data. So we decided to choose a subset consisting of all the features excluding the ones related to activities. This subset was called “No_activities”.

From this set of features without activities, we further constructed three subsets. The first one consisted only of the average daily values and their standard deviations (called “No_activities_avg_and_std_dev”). The second one consisted of the personalized patient values (called “No_activities_personalised”) and the third subset consisted only of the features that describe the changes between daily values (called “No_activities_changes”). We separated these subsets because they contain similar information, so they are redundant to some degree.

**Fig 4:**
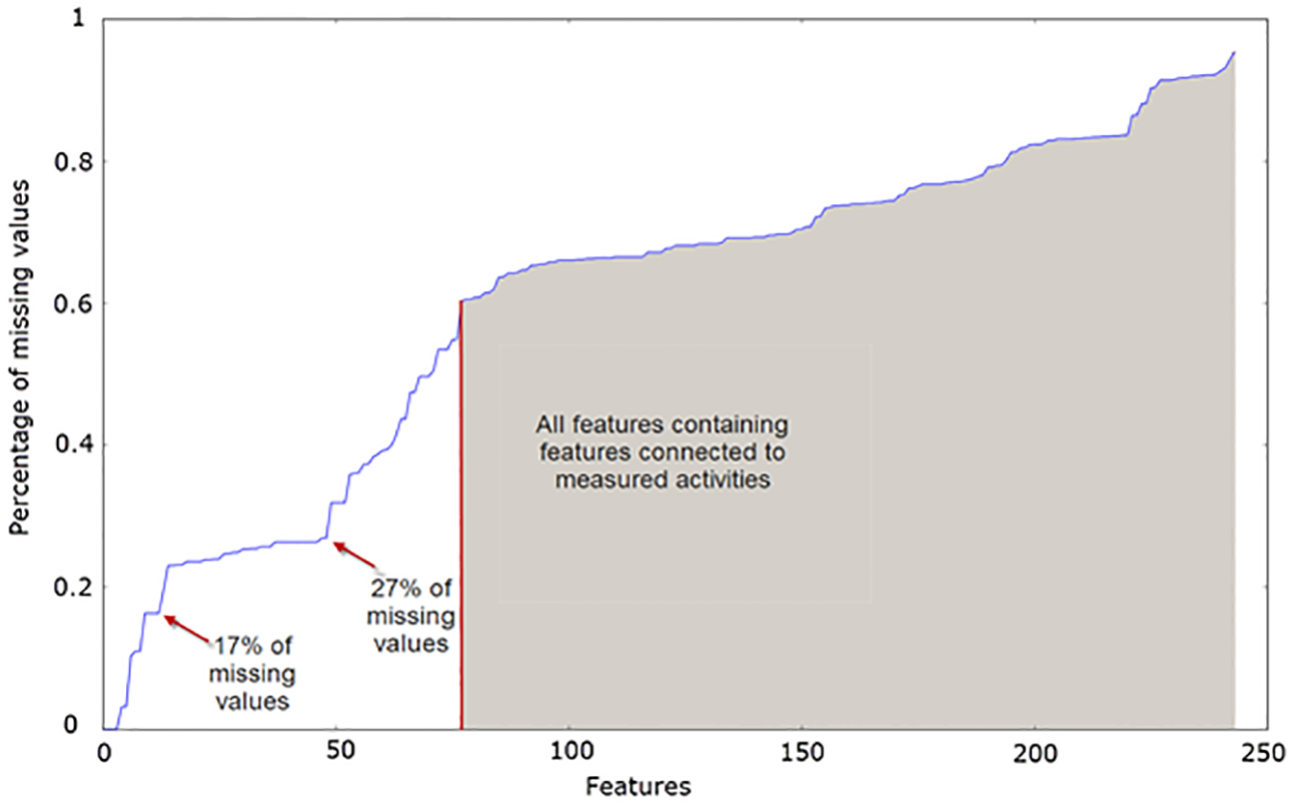
Features sorted by the percentage of missing values, with the two “knees” chosen as thresholds for feature selection.

Many of the features (not just the ones related to activities) had a lot of missing values. Fig 4 shows the features are sorted by the percentage of missing values. In the last two subsets we included only the features without too many missing values, because features with a lot of missing values can add noise to the predictions. To select the percentage of allowed missing values, we examined Fig 4 and spotted two “knees” in the graph, which are suitable to choose as thresholds. The subset thus included all the features that had less than 17% of missing values. The second subset included only the features having less than 27% of missing values. These feature subsets are called “No_sparse_features_0.17” and “No_sparse_features_0.27”.

### Imputation

After we defined the subsets, we performed imputation to fill in the missing data. We tried three different methods of imputation, k-nearest neighbors (kNN), Multiple Imputation by Chained Equations (MICE) and Singular Value Decomposition (SVD). Imputation with kNN [22] calculates missing feature values by finding the k most similar instances to the instance in question. The similarity is calculated from the other features as the sum of all differences between the instances in question and others. The missing value is imputed as the average of the k nearest neighbors’ values.

MICE imputation method [23] first fills the missing values with each feature’s mean values, but keeps these values marked. Then for each marked value the method creates a regression model to impute this value from the other features. After the missing value is thus imputed, it is used in regression model for imputing other missing values. After all the marked values are imputed, the process is repeated several times. The idea is that in each iteration, the regression models are better and the imputed values more accurate. In imputation with SVD, we used an established approach often applied to gene expression data [24]. We deceompose matrix A containing our dataset into a set of mutually orthogonal expression patterns that can be linearly combined to approximate the expression of all features in the data set as shown in the following equation. These patterns are further referred to as eigengenes.

$$A_{\left(m\times n\right)}=U_{\left(m\times m\right)}\sum_{{}_{\left(m\times n\right)}}V_{\left(n\times n\right)}^{T}$$

Matrix *V*^*T*^ now contains eigengenes, whose contribution to the expression in the eigenspace is quantified by corresponding eigenvalues on the diagonal of matrix Σ. We then identify the most significant eigengenes by sorting the eigengenes based on their corresponding eigenvalue. Once *k* most significant eigengenes from *V*^*T*^ are selected, we estimate a missing value *j* in gene *i* by first regressing this gene against the *k* eigengenes and then use the coefficients of the regression to reconstruct *j* from a linear combination of the *k* eigengenes. The j-th value of gene *i* and the j-th values of the *k* eigengenes are not used in determining these regression coefficients. It should be noted that SVD can only be performed on complete matrices; therefore we originally substitute row average for all missing values in matrix *A*, obtaining *A’*. We then utilize an expectation maximization method to arrive at the final estimate, as follows. Each missing value in *A’* is estimated using the above algorithm, and then the procedure is repeated on the newly obtained matrix, until the total change in the matrix falls below the empirically determined threshold of 0.01.

For every subset, we compared the results with these three imputation methods and without imputation. Based on the comparison we chose the most suitable imputation method or chose not to use the imputation if it did not bring any improvements.

### Class definitions

The patients that were participating in this study labelled how they felt with respect to their health in comparison to the previous day. Table 1 shows the frequency of the labels provided by the patients.

**Table 1:** Class definitions with number of instances for each.

Class name	Number of instances	Percentage of all instances
Feeling much worse than yesterday (1)	30	3
Feeling worse than yesterday (2)	73	7
Feeling the same as yesterday (3)	795	73
Feeling better than yesterday (4)	148	13
Feeling much better than yesterday (5)	40	4

If each of the five distinct feelings of health corresponded to one class, the differences between them would be too small to distinguish well between all classes. Therefore we decided for the purpose of data mining to use only two classes corresponding to good and bad feeling of health. Our first objective was to define the two classes in such a way that the difference between them would be large, so the models built with data mining would be as clear as possible. Since we wanted to learn the reasons for patients feeling better or worse, the cases where the patients were feeling the same as yesterday were not interesting and we did not include them in the data mining. According to all class definitions, the instances where the patients felt much worse or much better were assigned the bad or good class, because if a patient chose such a strong label, there was surely a real change in their feeling. Feeling worse or better than yesterday is sometimes hard for a patient to differentiate from feeling the same. The patients’ labels are subjective and probably inconsistent, so the different class definitions focus on the instances of feeling worse (2) and better (4). Table 2 shows the nine different class definitions used for data mining tasks. For each class definition there is a different number of instances that correspond to the definition. We can see that we have only 70 instances where a patient labeled his feeling with much worse or much better. Other definitions also include instances where the patient felt worse or better, in order to obtain a larger dataset to be able to train better prediction models.

**Table 2:** Class definitions with their name, the patients’ labels belonging to the classes bad and good, and the number of instances.

Class definition name	Class bad	Class good	Number of instances
1 vs. 5	Much worse	Much better	70
1, 2 vs. 4, 5	Much worse or worse	Much better or better	291
1, 2 x (2/3) vs. 4 x (2/3), 5	Much worse or worse two out of last three days	Much better or better two out of last three days	156
1, 2 x (2/4) vs. 4 x (2/4), 5	Much worse or worse two out of last four days	Much better or better two out of last four days	184
1, 2 x (2/5) vs. 4 x (2/5), 5	Much worse or worse two out of last five days	Much better or better two out of last five days	204
1, 2 x (3/4) vs. 4 x (3/4), 5	Much worse or worse three out of last four days	Much better or better three out of last four days	118
1, 2 x (3/5) vs. 4 x (3/5), 5	Much worse or worse three out of last five days	Much better or better three out of last five days	127
1, 2 x 2 vs. 4 x 2, 5	Much worse or worse two days in a row	Much better or better two days in a row	129
1, 2 x 3 vs. 4 x 3, 5	Much worse or worse three days in a row	Much better or better three days in a row	105

All the class definitions were used in data mining, although only the one that allowed to best distinguish between the two classes was utilized in the final steps.

### Data mining algorithms

For the data mining tasks we used several machine learning algorithms from the Weka suite [25]. The choice of the algorithms was based on a previous paper on the same topic [18]. In this paper we showed that Random forest (RF) [26], Naïve Bayes (NB) [27], Decision tree (DT) [28] and sequential minimal optimization (SMO) [29] obtained the best classification accuracies. Out of these four algorithms, DT and NB produce understandable classifiers, while RF and SMO typically excel at accuracy.

DT is a non-parametric supervised learning method used for classification and regression. It generates a tree-like classifier where leaves represent class labels and branches represent conjunctions of features that lead to those class labels. The paths from root to leaf represent classification rules. The goal is to create a classifier that predicts the value of a target variable by learning simple decision rules inferred from the data features. Since DT is a white box model, where the reasoning behind prediction is known, it is often used as a visual and analytical decision support tool.

NB is a simple probabilistic classifier based on applying Bayes' theorem with strong (naive) independence assumptions between the features. NB classifier assign class labels to problem instances, represented as vectors of feature values, where the class labels are drawn from some finite set. A NB classifier considers each of these features to contribute independently to the probability that an instance belongs to a specific class.

RF operates by constructing a multitude of DTs at training time and the outputting class represents the majority of the classes of the individual trees. RF with the insertion of randomness correct for decision trees' habit of overfitting to their training set.

SMO is used for training a support vector classifier (SVM) with solving the quadratic programming (QP) problem that arises during the training of SVM. An SVM classifier is a representation of the examples as points in space, mapped so that the examples of the separate classes are divided by a clear gap that is as wide as possible. New examples are then mapped into that same space and predicted to belong to a class based on which side of the gap they fall on.

To be able to estimate the quality of the obtained results, we also applied the majority classifier, which classifies all instances to the majority class. This classifier serves as a baseline that a reasonable classifier should exceed. All algorithms were run with default Weka parameter values.

### Experimental setup

For 9 different feature subset, for 9 class definition, for 4 data mining algorithm, and 4 imputation methods, we calculated the classification accuracy (CA). This was done by 10-fold cross-validation, and to minimize the effect of choosing a particular split of the data into 10 folds (subsets), the cross-validation was repeated 30 times with randomly chosen splits, and the CA calculated as the average over all the repetitions.

Since there are 1296 combinations of data mining algorithms, feature subsets, imputation methods and class definitions, we were not able to efficiently present the results for all possible combinations. So, we decided to first calculate the accuracies for all the combinations. Afterwards, we evaluated each aspect in turn: which data mining algorithm perform best, whether imputation is needed, and how feature subsets and class definitions compare.

Finally, we built decision tree classifiers and studied the relations in these classifiers through the lens of expert knowledge on CHF. We annotated the relations as (i) correct, (ii) wrong, (iii) novel, (iv) unrelated or (v) unknown, which was based on standard medical knowledge [10, 30–37]. The novel relations could offer new insights into how to improve PROs among CHF patients.

## Results

We started the analysis of our dataset by comparing different feature subsets with different data mining algorithms. The result is a big table (presented in S1 Table) presenting the average classification accuracies over all the class definitions for each of the four selected data mining algorithm and each of the 36 subsets, including subsets obtained with imputation. The best result was obtained with the RF on the *No_sparse_features_0*.*17_kNN* subset. Additionally, we calculated the average CA for every data mining algorithm over all the subsets. The results are presented in Table 3 and the highest accuracy is marked in bold.

**Table 3:** The classification accuracy for each tested data mining algorithm, averaged over all the class definitions and subsets.

Data mining algorithms	Random forest	Decision tree	Naive Bayes	SMO	Majority
Average CA over all class definitions and subsets	**75.9%**	74.0%	69.1%	70.0%	66.9%

We can see that the RF performs best, and that the DT is clearly second while being a white box model that enables us to understand its predictions. We performed statistical tests to determine if RF and DT are statistically significantly better than the NB and SMO. For the comparisons we used the Wilcoxon signed ranked test [38], which is considered the most appropriate choice when comparing two or more classifiers over multiple data subsets because it assumes some but limited commensurability, and it is also safer than parametric tests since it does not assume normal distributions or homogeneity of variance [19, 39]. As such, it can be applied to classification accuracies (as in our case), error ratios or any other measure for evaluation of classifiers. Empirical results suggest that it is also stronger than other tests particularly when comparing a pair of classifiers. We pairwise compared RF with NB and SMO and DT with NB and SMO on all 36 subsets. The results (provided in the S1 Table) show that both RF and DT are statistically significantly better than NB and SMO over all data subsets. So, for further calculations we decided that we will only use only RF and DT. Additionally, we also statistically compared RF and DT and the results obtained by the RF proved to be statistically significantly better.

Next we wanted to determine if the imputation helps CA or not. So, we compared feature subsets without imputation with feature subsets obtained with three different imputation methods. The CA for each imputation approach for every feature subset is presented in S2–S5 Tables. The CA averaged over all nine subsets per each imputation approach for RF and DT is presented in Table 4. The highest accuracy for each data mining algorithms is in bold.

**Table 4:** The classification accuracy for the various imputation approaches, averaged over all the class definitions and subsets.

Algorithms / Imputation method	Random Forest	Decision tree	Average RF and DT
No imputation	**77.36**	**75.75**	**76.55**
Imputation with kNN	76.07	74.66	75.37
Imputation with MICE	74.29	72.54	73.41
Imputation with SVD	75.78	72.92	74.35

We can see that the best average CA is obtained without the use of imputation. The most likely reason is the large amount of missing data. Due to the missing data the imputations are not accurate and thus add additional noise to the data. But nonetheless the subset that obtained the best CA over all class definitions and data mining algorithms is *No_sparse_features_0*.*17_kNN*, obtained with the kNN imputation. This is the only subset where the imputation improves the results, most likely because all the features with many missing values are removed. Because the best subset is obtained with the kNN imputation, but otherwise the use of imputation makes the results worse, we decided that for further calculations we will use the nine subsets without imputation and additionally the subset obtained with the kNN imputation on the feature subset *No_sparse_features_0*.*17*. The CA for each of the retained subsets for RF and DT is presented in Table 5. The highest accuracy for each data mining algorithms is in bold.

**Table 5:** The classification accuracy for various feature subsets, averaged over all the class definitions.

Algorithms /Subsets	Random forest	Decision tree	Average RF and DT
All	74.97	75.58	75.27
CFS_feature_selection	80.32	78.96	79.64
Expert_selection	80.58	79.12	79.85
No_activities	77.34	77.18	77.26
No_activities_avg_and_std_dev	79.65	75.36	77.51
No_activities_changes	67.97	70.79	69.38
No_activities_personalised	72.04	67.72	69.88
No_sparse_features_0.17	83.11	78.46	80.79
No_sparse_features_0.27	80.21	78.56	79.39
No_sparse_features_0.17_kNN	**84.21**	**81.67**	**82.94**
**Average**	78.04	76.34	77.19

As indicated before, we can see that the *No_sparse_features_0*.*17_kNN* subset returns the best results with both RF and DT. The CAs for different class definitions for this subset are presented in Table 6. The highest accuracy for each data mining algorithms is in bold.

**Table 6:** The classification accuracy for all nine class definitions on the *No_sparse_features_0*.*17_kNN* subset.

Algorithms / Class definitions	Random forest	Decision tree
Class 1 vs. 5	80.48	77.71
Class 1, 2 vs. 4, 5	71.35	69.82
Class 1, 2 x (2/3) vs. 4 x (2/3), 5	82.94	78.14
Class 1, 2 x (2/4) vs. 4 x (2/4), 5	79.51	76.89
Class 1, 2 x (2/5) vs. 4 x (2/5), 5	78.95	77.78
Class 1, 2 x (3/4) vs. 4 x (3/4), 5	**86.78**	**86.10**
Class 1, 2 x (3/5) vs. 4 x (3/5), 5	85.18	83.04
Class 1, 2 x 2 vs. 4 x 2, 5	83.45	81.54
Class 1, 2 x 3 vs. 4 x 3, 5	86.46	83.47
**Average**	81.68	79.39

**Fig 5:**
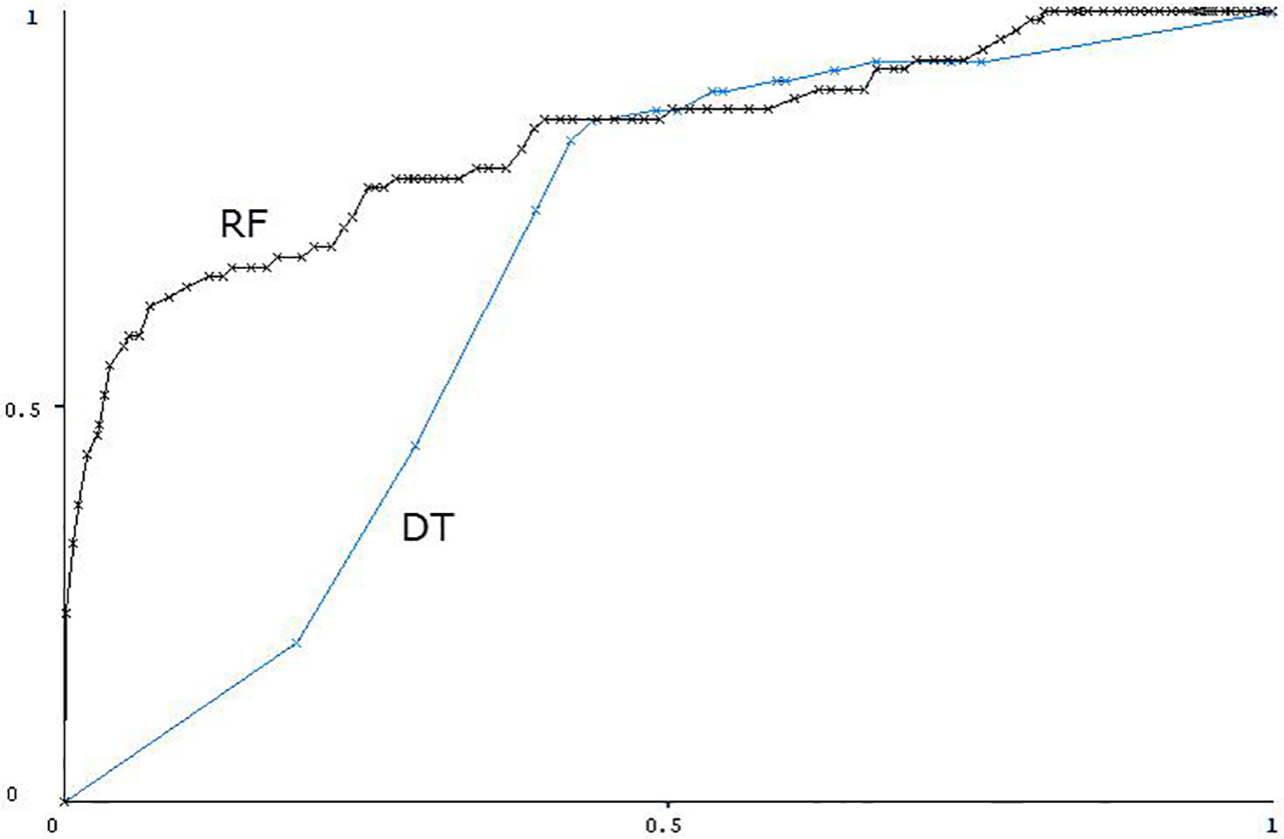
The ROC curves for RF and DT for class definition Class 1, 2 x (3/4) vs. 4 x (3/4), 5 on the *No_sparse_features_0*.*17_kNN* subset.

The average CAs in Table 6 are quite close to each other, which shows that the carefully chosen class definitions are not the reason for good results. According to all the definitions, the instances when the patients felt much worse (value 1) than the previous day were included in the bad class, and the instances when the patients felt much better (value 5) in the good class. We can see that the accuracy is the lowest when all the instances when the patients felt worse (2) or better (4) for just one day were included additionally (*Class 1*,*2 vs*. *4*,*5*). Moderately high CAs were achieved when the instances when the patients felt worse or better for two out of the last two to five days were included (*Class 1*, *2 x (2/3) vs*. *4 x (2/3)*, *5; Class 1*, *2 x (2/4) vs*. *4 x (2/4)*, *5; Class 1*, *2 x (2/5) vs*. *4 x (2/5)*, *5; Class 1*, *2 x 2 vs*. *4 x 2*, *5*). Such instances represented a more marked worsening or improvement of health, and were thus easier to distinguish. The results were similar when no instances when the patients felt worse or better were included (*Class 1 vs*. *5*). These instances were the easiest to distinguish, but there were relatively few, so the dataset was too small. The best CAs were achieved when the instances when the patients felt worse or better for three out of the last three to five days were included (*Class 1*, *2 x (3/4) vs*. *4 x (3/4)*, *5; Class 1*, *2 x (3/5) vs*. *4 x (3/5)*, *5; Class 1*, *2 x 3 vs*. *4 x 3*, *5*), out of which the class definition *Class 1*, *2 x (3/4) vs*. *4 x (3/4)*, *5* distinguished best between patients feeling bad and feeling good. For this class definition on the *No_sparse_features_0*.*17_kNN* subset a calculated the ROC curve [40] for RF presented in Fig 5. The AUC [41] is 0.835. For the comparison we also calculated the ROC curve for the DT (Fig 5). The AUC for the DT is 0.674.

**Fig 6:**
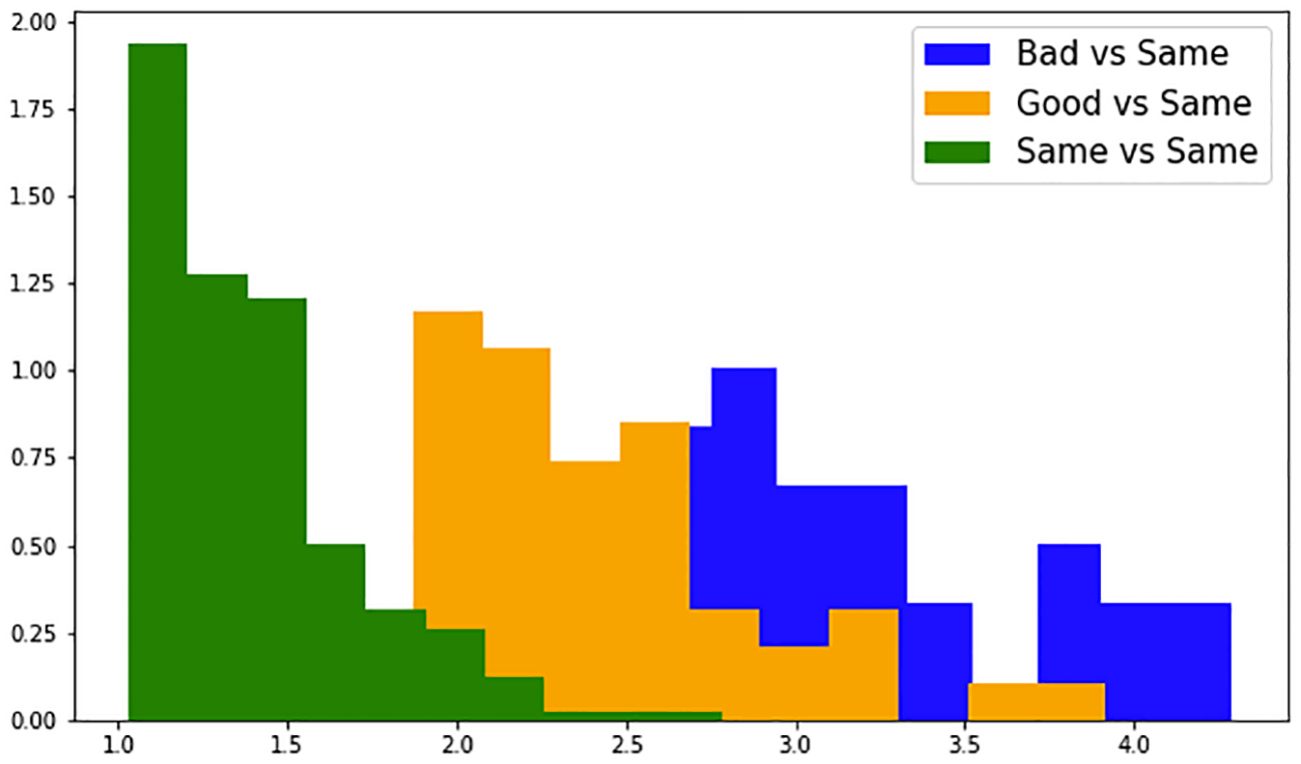
Probability densities of the average feature differences between the classes feeling the same as yesterday, good and bad.

Since all the class definitions exclude the PRO of feeling the same as yesterday (3), we checked whether the instances in the good and bad classes are indeed distinct from the instances when the patients felt the same as yesterday. We used the best subset *No_sparse_features_0*.*17_kNN*, and first standardized the features (subtracted the mean and divided by the standard deviation). For each pair of instances when the patients felt the same as yesterday, we then computed the average difference between all the features–this provided a measure of variation between the days when feeling the same as yesterday. Finally, we did the same for each pair of instances where one instance corresponded to feeling the same as yesterday, and the other belonged to the class good or bad according to the best class definition *1*, *2 x (3/4) vs*. *4 x (3/4)*, *5* –this provided a measure of difference between days when feeling the same as yesterday and days when feeling good or bad. Fig 6. presents probability densities of these differences, showing that the variation between days when feeling the same as yesterday is smaller than the difference between days when feeling the same as yesterday and days when feeling good or bad. The features are from the subset *No_sparse_features_0*.*17_kNN*, and the class is defined as *1*, *2 x (3/4) vs*. *4 x (3/4)*, *5*. The differences are divided in 10 bins, and the counts the bins are normalized so that the area under the histogram for each difference sums to 1.

**Fig 7:**
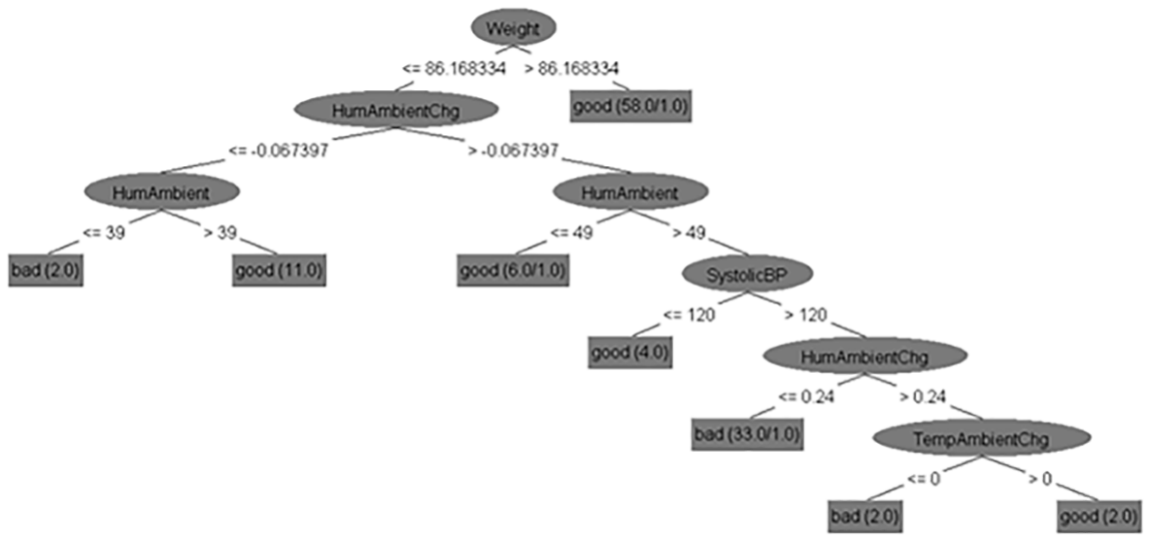
Decision tree for the class definition *Class 1*, *2 x (3/4) vs*. *4 x (3/4)*, *5* built on the *No_sparse_features_0*.*17_kNN subset*.

**Fig 8:**
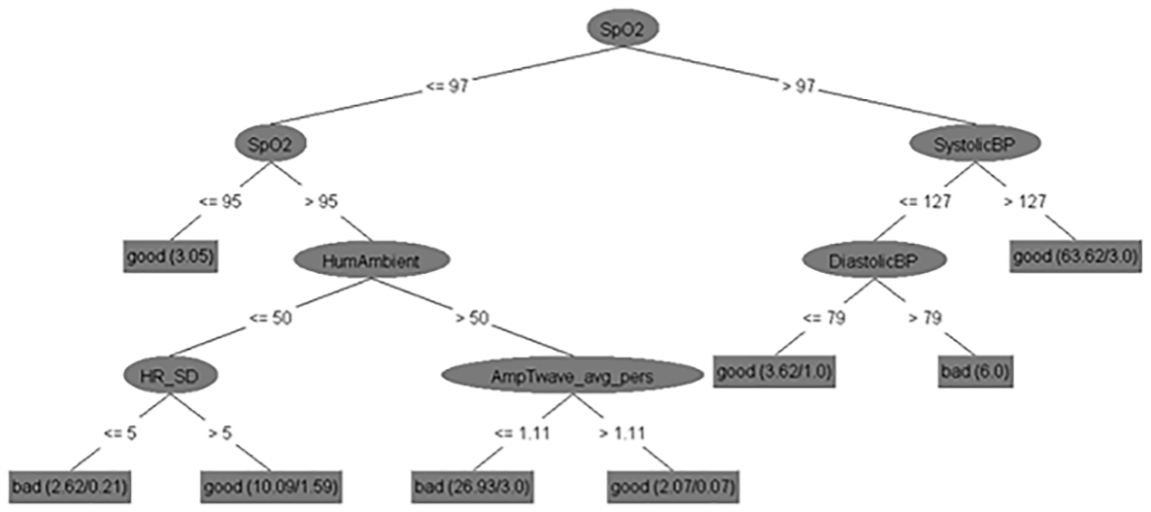
Decision tree for the class definition *Class 1*, *2 x (3/4) vs*. *4 x (3/4)*, *5* built on the *Expert_selection subset*.

After having established how high CA accuracy distinguishing bad and good feeling of health of CHF patients can be achieved by mining our data, we studied decision trees to understand which features affect the feeling of health. We present two trees here, while the others are in S1–S10 Figs. The trees are presented for the class definition *Class 1*, *2 x (3/4) vs*. *4 x (3/4)*, *5*. Fig 7 presents the decision tree obtained on the *No_sparse_features_0*.*17_kNN* subset that returned the best CA of 86% and Fig 8 shows the decision tree obtained on the *Expert_selectio*n feature subset with the CA of 83%.

All the obtained decision tree classifiers (not only those two in Figs 4 and 5) were systematically analyzed to see how they match the current medical knowledge and whether they contain new knowledge. Table 7 presents the relations that occur in the ten classifiers more than once (the list of all the relations is provided in S6 Table). They were mostly studied qualitatively, which means that we considered whether a high or low value of each parameter should be associated with good or bad feeling. The exact cut-off values were considered less important as they are heavily dependent on the patients and their environment (e.g., we can expect different humidities and the patients’ responses to them in our Italian and British data compared to the published data from Hong Kong of Australia [42, 43]). Most tree nodes were considered in isolation, which means that we studied the parameter in each node and the distribution of the classes in the subtrees rooted in that node’s descendants. In some cases, however, the relations to nodes above were considered as well–we used expert judgement in this. The table provides references that support the relations when we could find them, while further interpretation is presented in the Discussion section.

**Table 7:** An overview of the relations between the features and the feeling of health occurring more than once in the decision trees for all the feature subsets.

Relation	Cut-off	Occurrences	Supporting references
Ambient humidity (avg and chg) small: good, large: bad	49 to 50 and –0.13 to –0.0674	12	In favor [42, 43]
Ambient humidity small: bad, large: good	39	3	In favor [43]
Weight small: bad, large: good	86.2 to 87	7	
Weight change small: good, large: bad	0	2	In favor [32]
Systolic BP (avg and pers) small: good, large: bad	120 and 1.10	6	Against [44]
Systolic BP (avg and chg) small: bad, large: good	127 and –0.11	2	In favor [44]
Humidity ratio (avg and pers) small: good, large: bad	0.74 to 0.75	4	In favor [46, 47]
Ambient temperature (avg and chg) small: bad, large: good	24 and 0	3	In favor [32, 46, 47]
QRS duration (pers and chg) large: good, small: bad	1.03 and 0.01	3	Against [49]
SpO2 small: bad, large: good	97	2	In favor [51]
SpO2 small: good, large: bad	95	2	Against [51]

## Discussion

The first important finding of this paper is that objective telemonitored parameters can predict subjective feeling of health. This result is the first one, to our knowledge, to connect objective parameters to PROs, and thus enables the possibility of using PROs as primary end-points in future trials. The highest accuracies achieved using machine learning were over 86%, and the AUC was 0.79 (which was obtained by RF, see Table 6, Fig 3). This is comparable with the AUC of 0.82 from related work [14] achieved for the prediction of hospitalizations, which appears to be an easier problem because hospitalization are typically preceded by more pronounced physiological changes. While two significantly different experiments cannot be directly compared, we can still speculate that the parity between the results is due to the richer features enabled by wearable devices in our case.

The highest accuracy for predicting the feeling of health was achieved by carefully defining the class (what it means to feel bad or good). However, the accuracies for all the considered class definitions were above 79% with one exception. This shows that telemonitored parameters can predict the feeling of health under a relatively wide range of conditions, confirming the validity of this finding. The exception was the definition that required the weakest expression of bad or good feeling of health, which shows that either the patients’ subjective experience is more sensitive than what intelligent computer methods can extract from telemonitored parameters, that patients cannot highly reliably distinguish feeling bad or good for reasons of health vs. for other reasons, or that there are simply some inconsistentcies and biases in the PROs.

The other important findings of the paper concern the relations in the DTs, which provide an insight into which features make CHF patients feel bad or good. The accuracy of DTs is admittedly lower than of RFs, so RF should be used when the objective is to simply predict the feeling of health, but for the analysis of the relations, DTs are most suitable since they are understandable and only 2.29 percentage point less accurate than RFs on average. The most often occurring relation in our DTs concerns ambient humidity: when it is high or increasing, the patients feel bad, and sometimes when it is low (below 39%), they also feel bad. A high (and low) humidity is known [42, 43] (albeit not widely accepted) to be problematic for CHF patients, so these two relations are on one hand reasonable, and on the other suggest ambient humidity should be investigated prospectively among CHF patients.

A very prominent relation is also that the patients weighing more than 86.2 to 87 kg feel good. Since a relatively modest number of patients were included in the study, one patient could skew the results. Patient 56 (see Fig 2) was such a case–he/she labeled the feeling of health as good much more often than other patients, and was at the same time heavier than others. A positive weight change is associated with feeling bad, which is expected as it signifies fluid retention–a common problem in CHF patients [32].

The relations concerning systolic BP are conflicting, but large (above 120 mmHg) or increasing BP is more often associated with feeling bad, which is unusual, as low BP is more often a problem in CHF than high BP [44]. However, in these unusual cases the BP relation splits 2–4 instances of feeling good from a much larger number of instances of feeling bad, so they are probably coincidental (one example of this is in Fig 4). The opposite relation, which is expected in CHF patients, is shown in Fig 5. The right side of the decision tree states that systolic BP above 127 mmHg leads to feeling good, which is reasonable since 127 mmHg is not too high in itself. Systolic BP under 127 mmHg–as long as diastolic BP is under 79 mmHg–is also good, while systolic and diastolic pressure being close to each other, which indicates poor heart function in CHF patients, lead to feeling bad [45].

Humidity ratio is the ratio between the skin and ambient humidity, so it indicates sweating corrected for the ambient humidity. Since the study took place in the cold part of the year, sweating is probably not due to high ambient temperatures. It can thus be explained as a problem with the sympathetic neural system and as such may deserve further study [46, 47].

High and increasing ambient temperature is associated with feeling good. This can be explained by CHF patients feeling cold in the extremities due to poor blood circulation [48], and the literature also reports increased hospitalizations and mortality during cold weather [32]. Cold extremities are also related to sympathetic over-activation [46, 47]. The effect of ambient temperature is not often studied in CHF patients, so this finding is interesting and may deserve further investigation.

Our decision trees suggest that large or increasing QRS-interval duration leads to feeling good, which is difficult to interpret. QRS prolongation has been shown to signify worse outcomes in CHF patients [49], which seems to contradict our findings. However, unlike the literature on QRS prolongation, we studied short-term changes in QRS-interval duration, which may be related to QRS fragmentation [50], erroneous ECG analysis by the Falcon algorithm, or some other phenomenon. More investigations are needed in this area to clarify the relation between QRS duration, whether related to QRS fragmentation or not, and PROs.

We have conflicting relations concerning SpO2, but high SpO2 leading to feeling good (as expected [51]) occurs in considerably more instances than the opposite. The relation of low SpO2 leading to feeling good is thus probably spurious–the patients felt good for reasons unrelated to SpO2.

In Fig 7 it is also interesting that high standard deviation of heart rate is good, which may suggest that the heart can adapt to the demands of the users’ activities. This is similar to the finding that a large range of heart rate leads to fewer hospitalizations and mortality [52]. The relation appears only in this decision tree, which is why it is not included in Table 7.

Finally, there is a relation that is interesting because of its absence: low heart rate is generally considered an indicator of good outcomes in CHF, but is not present in any decision tree. The average heart rate among the instances of feeling good [53, 54] is in fact substantially lower than of feeling bad (68 vs. 74 beats per minute), but the machine-learning algorithm still found other relations to produce more accurate decision trees.

## Conclusion

This paper presents the results of mining the data collected during a telemonitoring study of CHF patients. The study included 24 patients, who used a range of wearable and other sensing devices, and provided information on how they felt each day. Such PROs are increasingly seen as crucial for the management of CHF and other incurable diseases, where the quality of life is an important goal independent of “hard” outcomes such as mortality and hospitalizations. We constructed a large number of features from the telemonitored parameters and built classifiers that predicted how the patients feel. To this end, we systematically analyzed various data mining algorithms, feature imputation methods, feature subsets and the definitions of the class to predict. We achieved prediction accuracies up to 86% and AUC up to 0.83, demonstrating that the telemonitored parameters contain valuable information about the patient’s health-related quality of life.

We also studied the relations contained in the decision tree classifiers to understand the effect of the telemonitored parameters on the quality of life. We found several relations that agree with the current medical knowledge: systolic BP above 127 mmHg and SpO2 above 97% often lead to feeling good, while increasing weight (probably due to fluid retention) leads to feeling bad. This can be seen as a confirmation of the validity of our data and the data-mining approach. We also found some relations that are known but less well established: high (and low) ambient humidity, low temperature and sweating lead to feeling bad. These findings can contribute to better management of CHF in the future, since at least the humidity and temperature are not difficult to monitor and control. Some relations in the classifiers disagree with the current medical knowledge, which is most likely due to having insufficient data.

While the size of Chiron telemonitoring study in terms of the number of patients is modest, the level of detail is very high due to the large number of monitored parameters, including continuous ECG, as well as the additional parameters extracted via advanced ECG and activity analysis. Because of this, we decided for an exploratory approach to the analysis of the collected data using data mining methods. Such an approach can provide a broader range of insights than the more traditional statistical approaches, albeit with fewer guarantees regarding significance. The findings reported in this paper will serve as inputs to another–more extensive–study of CHF patients planned in the ongoing HeartMan project.

The predictive models presented in this paper are interesting not only for the insights into the relations between the telemonitored parameters and the quality of life, but also as a foundation for an intelligent system that can provide advice on how to improve CHF patients’ quality of life. To do so, we would first have to identify parameters that are modifiable by the patients (e.g., temperature, humidity and exercise-related parameters). Afterwards, we could use computational methods to identify parameter values that are expected to lead to an improved feeling of health, and advise the patients to modify the relevant parameters accordingly. This is the subject of our ongoing research.

## Supporting information

S1 Fig Decision tree for the class definition *Class 1*, *2 x (3/4) vs*. *4 x (3/4)*, *5* built on the *No_sparse_features_0*.*17_kNN subset*. (PDF)

S2 Fig Decision tree for the class definition *Class 1*, *2 x (3/4) vs*. *4 x (3/4)*, *5* built on the *Expert_selection subset*. (PDF)

S3 Fig Decision tree for the class definition Class 1, 2 x (3/4) vs. 4 x (3/4), 5 built on the No_sparse_features_0.17 subset. (PDF)

S4 Fig Decision tree for the class definition Class 1, 2 x (3/4) vs. 4 x (3/4), 5 built on the No_sparse_features_0.27 subset. (PDF)

S5 Fig Decision tree for the class definition Class 1, 2 x (3/4) vs. 4 x (3/4), 5 built on all features. (PDF)

S6 Fig Decision tree for the class definition Class 1, 2 x (3/4) vs. 4 x (3/4), 5 built on the CFS_feature_selection subset. (PDF)

S7 Fig Decision tree for the class definition Class 1, 2 x (3/4) vs. 4 x (3/4), 5 built on the No_activities subset. (PDF)

S8 Fig Decision tree for the class definition Class 1, 2 x (3/4) vs. 4 x (3/4), 5 built on the No_activities_avg_and_std_dev subset. (PDF)

S9 Fig Decision tree for the class definition Class 1, 2 x (3/4) vs. 4 x (3/4), 5 built on the No_activities_changes subset. (PDF)

S10 Fig Decision tree for the class definition Class 1, 2 x (3/4) vs. 4 x (3/4), 5 built on the No_activities_personalised subset. (PDF)

S1 Table The classification accuracy for each feature subsets and data mining algorithm averaged over all the class definitions. (DOCX)

S2 Table The classification accuracy for each feature subset and data mining algorithms, averaged over all the class definitions, without imputation. (DOCX)

S3 Table The classification accuracy for each feature subset and data mining algorithms, averaged over all the class definitions, with kNN imputation. (DOCX)

S4 Table The classification accuracy for each feature subset and data mining algorithms, averaged over all the class definitions, with MICE imputation. (DOCX)

S5 Table The classification accuracy for each feature subset and data mining algorithms, averaged over all the class definitions, with SVD imputation. (DOCX)

S6 Table Relations obtained from the ten decision trees in S1–S10 Figs. with their types and cut-off values. The references in table are provided only for relation types not discussed in the body of the paper.
(DOCX)

S1 Dataset The entire telemonitored physiological data set obtained from the study as described in the paper. (CSV)

## References

[1] Cook C, Cole G, Asaria P, Jabbour R, Francis DP. The annual global economic burden of heart failure. International journal of cardiology. 2014;171(3):368–76. doi: 10.1016/j.ijcard.2013.12.028 24398230 10.1016/j.ijcard.2013.12.028

[2] Bashi N, Karunanithi M, Fatehi F, Ding H, Walters D. Remote monitoring of patients with heart failure: an overview of systematic reviews. Journal of medical Internet research. 2017;19(1). 10.2196/jmir.6571 PMC5291866 28108430

[3] Kotb A, Cameron C, Hsieh S, Wells G. Comparative effectiveness of different forms of telemedicine for individuals with heart failure (HF): a systematic review and network meta-analysis. PloS one. 2015;10(2). 10.1371/journal.pone.0118681 PMC4340962 25714962

[4] Sousa C, Leite S, Lagido R, Ferreira L, Silva‐Cardoso J, Maciel MJ. Telemonitoring in heart failure: A state‐of‐the‐art review. Revista Portuguesa de Cardiologia (English Edition). 2014;33(4):229–39. 10.1016/j.repc.2013.10.013 24830309

[5] Ledwidge MT, O'hanlon R, Lalor L, Travers B, Edwards N, Kelly D, et al Can individualized weight monitoring using the HeartPhone algorithm improve sensitivity for clinical deterioration of heart failure? European journal of heart failure. 2013;15(4):447–55. doi: 10.1093/eurjhf/hfs186 23204211 10.1093/eurjhf/hfs186

[6] US Department of Health and Human Services FDA Center for Drug Evaluation and Research, US Department of Health and Human Services FDA Center for Biologics Evaluation and Research, & US Department of Health and Human Services FDA Center for Devices and Radiological Health. (2006). Guidance for industry: patient-reported outcome measures: use in medical product development to support labeling claims: draft guidance. Health and Quality of Life Outcomes, 4, 1–20. 17034633 10.1186/1477-7525-4-79 PMC1629006

[7] Anker SD, Agewall S, Borggrefe M, Calvert M, Jaime Caro J, Cowie MR, et al The importance of patient-reported outcomes: a call for their comprehensive integration in cardiovascular clinical trials. European heart journal. 2014;35(30):2001–9. doi: 10.1093/eurheartj/ehu205 24904027 10.1093/eurheartj/ehu205

[8] Organization WH. The world health report 2002: reducing risks, promoting healthy life: World Health Organization; 2002. 10.1080/1357628031000116808 14741909

[9] Ezzati M, Vander Hoorn S, Lopez AD, Danaei G, Rodgers A, Mathers CD, et al Comparative quantification of mortality and burden of disease attributable to selected risk factors. Global burden of disease and risk factors. 2006;2:241–396. 21250375

[10] Puddu PE, D’Ambrosi A, Scarparo P, Centaro E, Torromeo C, Schiariti M, et al A clinician’s view of next-generation remote healthcare system Systems design for remote healthcare: Springer; 2014 p. 1–30.

[11] Gnanasakthy A, Mordin M, Clark M, DeMuro C, Fehnel S, Copley-Merriman C. A review of patient-reported outcome labels in the United States: 2006 to 2010. Value in health. 2012;15(3):437–42. doi: 10.1016/j.jval.2011.11.032 22583453 10.1016/j.jval.2011.11.032

[12] Grady KL, Stevenson LW, Pagani FD, Teuteberg J, Pamboukian SV, Birks E, et al Beyond survival: Recommendations from INTERMACS for assessing function and quality of life with mechanical circulatory support. Elsevier; 2012. 10.1016/j.healun.2012.08.020 23010671

[13] Tripoliti EE, Papadopoulos TG, Karanasiou GS, Naka KK, Fotiadis DI. Heart Failure: Diagnosis, Severity Estimation and Prediction of Adverse Events Through Machine Learning Techniques. Computational and structural biotechnology journal. 2017;15:26–47. doi: 10.1016/j.csbj.2016.11.001 27942354 10.1016/j.csbj.2016.11.001 PMC5133661

[14] Koulaouzidis G, Iakovidis D, Clark A. Telemonitoring predicts in advance heart failure admissions. International journal of cardiology. 2016;216:78–84. doi: 10.1016/j.ijcard.2016.04.149 27140340 10.1016/j.ijcard.2016.04.149

[15] Kang Y, McHugh MD, Chittams J, Bowles KH. Utilizing home health care electronic health records for telehomecare patients with heart failure: a decision tree approach to detect associations with rehospitalizations. Computers, informatics, nursing: CIN. 2016;34(4):175 doi: 10.1097/CIN.0000000000000223 26848645 10.1097/CIN.0000000000000223 PMC4950866

[16] Fisher R, Smailagic A, Simmons R, Mizobe K, editors. Using Latent Variable Autoregression to Monitor the Health of Individuals with Congestive Heart Failure. Machine Learning and Applications (ICMLA), 2016 15th IEEE International Conference on; 2016: IEEE.

[17] Pecchia L, Melillo P, Bracale M. Remote health monitoring of heart failure with data mining via CART method on HRV features. IEEE Transactions on Biomedical Engineering. 2011;58(3):800–4. doi: 10.1109/TBME.2010.2092776 21078568 10.1109/TBME.2010.2092776

[18] Luštrek M, Cvetković B, Bordone M, Soudah E, Cavero C, Rodríguez JM, et al., editors. Supporting clinical professionals in decision-making for patients with chronic diseases. Proc 15th Int Multiconf Inf Soc; 2012.

[19] Luštrek M, Cvetković B, Kozina S, editors. Energy expenditure estimation with wearable accelerometers. Circuits and Systems (ISCAS), 2012 IEEE International Symposium on; 2012: IEEE.

[20] Sun Y, Wu D, editors. A relief based feature extraction algorithm. Proceedings of the 2008 SIAM International Conference on Data Mining; 2008: SIAM.

[21] Hall MA. Correlation-based feature selection for machine learning. 1999.

[22] Acuna E, Rodriguez C. The treatment of missing values and its effect on classifier accuracy. Classification, clustering, and data mining applications. 2004:639–47.

[23] Buuren S, Groothuis-Oudshoorn K. mice: Multivariate imputation by chained equations in R. Journal of statistical software. 2011;45(3).

[24] Troyanskaya O, Cantor M, Sherlock G, Brown P, Hastie T, Tibshirani R, et al Missing value estimation methods for DNA microarrays. Bioinformatics. 2001;17(6):520–5. 11395428 10.1093/bioinformatics/17.6.520

[25] Hall M, Frank E, Holmes G, Pfahringer B, Reutemann P, Witten IH. The WEKA data mining software: an update. ACM SIGKDD explorations newsletter. 2009;11(1):10–8.

[26] Liaw A, Wiener M. Classification and regression by randomForest. R news. 2002;2(3):18–22.

[27] Rish I, editor An empirical study of the naive Bayes classifier IJCAI 2001 workshop on empirical methods in artificial intelligence; 2001: IBM.

[28] Friedl MA, Brodley CE. Decision tree classification of land cover from remotely sensed data. Remote sensing of environment. 1997;61(3):399–409.

[29] Platt J. Sequential minimal optimization: A fast algorithm for training support vector machines. 1998.

[30] Donnelly M, Paggetti C, Nugent C, Mokhtari M. Impact Analysis of Solutions for Chronic Disease Prevention and Management: 10th International Conference on Smart Homes and Health Telematics, ICOST 2012, Artimino, Tuscany, Italy, June 12–15, Proceedings: Springer; 2012.

[31] Bonfiglio S, editor Fostering a Continuum of Care. ICOST; 2012: Springer.

[32] Puddu PE, Morgan JM, Torromeo C, Curzen N, Schiariti M, Bonfiglio S, editors. A clinical observational study in the CHIRON project: rationale and expected results. International Conference on Smart Homes and Health Telematics; 2012: Springer.

[33] Luštrek M, Somrak M. Mining Telemonitoring Data From Congestive-Heart-Failure Patients.

[34] Puddu PE, Menotti A. Artificial neural network versus multiple logistic function to predict 25-year coronary heart disease mortality in the Seven Countries Study. European Journal of Cardiovascular Prevention & Rehabilitation. 2009;16(5):583–91. 19602982 10.1097/HJR.0b013e32832d49e1

[35] Puddu PE, Menotti A. Artificial neural networks versus proportional hazards Cox models to predict 45-year all-cause mortality in the Italian Rural Areas of the Seven Countries Study. BMC medical research methodology. 2012;12(1):100. 22824187 10.1186/1471-2288-12-100 PMC3549727

[36] Piras P, Teresi L, Gabriele S, Evangelista A, Esposito G, Varano V, et al, editors. Systo-Diastolic LV Shape Analysis by Geometric Morphometrics and Parallel Transport Highly Discriminates Myocardial Infarction. International Workshop on Statistical Atlases and Computational Models of the Heart; 2015: Springer.

[37] Mazomenos EB, Biswas D, Acharyya A, Chen T, Maharatna K, Rosengarten J, et al A low-complexity ECG feature extraction algorithm for mobile healthcare applications. IEEE journal of biomedical and health informatics. 2013;17(2):459–69. doi: 10.1109/TITB.2012.2231312 23362250 10.1109/TITB.2012.2231312

[38] Wilcoxon F. Individual comparisons by ranking methods. Biometrics bulletin. 1945;1(6):80–3. 18903631

[39] Demšar J. Statistical comparisons of classifiers over multiple data sets. Journal of Machine learning research. 2006;7(Jan):1–30.

[40] Hanley JA, McNeil BJ. The meaning and use of the area under a receiver operating characteristic (ROC) curve. Radiology. 1982;143(1):29–36. doi: 10.1148/radiology.143.1.7063747 7063747 10.1148/radiology.143.1.7063747

[41] Huang J, Ling CX. Using AUC and accuracy in evaluating learning algorithms. IEEE Transactions on knowledge and Data Engineering. 2005;17(3):299–310.

[42] Goldie J, Sherwood SC, Green D, Alexander L. Temperature and humidity effects on hospital morbidity in Darwin, Australia. Annals of global health. 2015;81(3):333–41. doi: 10.1016/j.aogh.2015.07.003 26615068 10.1016/j.aogh.2015.07.003

[43] Goggins WB, Chan EY. A study of the short-term associations between hospital admissions and mortality from heart failure and meteorological variables in Hong Kong: Weather and heart failure in Hong Kong. International journal of cardiology. 2017;228:537–42. doi: 10.1016/j.ijcard.2016.11.106 27875731 10.1016/j.ijcard.2016.11.106

[44] Lee TT, Chen J, Cohen DJ, Tsao L. The association between blood pressure and mortality in patients with heart failure. American heart journal. 2006;151(1):76–83. doi: 10.1016/j.ahj.2005.03.009 16368295 10.1016/j.ahj.2005.03.009

[45] Jackson CE, Castagno D, Maggioni AP, Køber L, Squire IB, Swedberg K, et al Differing prognostic value of pulse pressure in patients with heart failure with reduced or preserved ejection fraction: results from the MAGGIC individual patient meta-analysis. European heart journal. 2015;36(18):1106–14. doi: 10.1093/eurheartj/ehu490 25616644 10.1093/eurheartj/ehu490

[46] Balmain BN, Sabapathy S, Jay O, Adsett J, Stewart GM, Jayasinghe R, et al Heart Failure and Thermoregulatory Control: Can Patients with Heart Failure Handle the Heat? Journal of Cardiac Failure. 2017. 10.1016/j.cardfail.2017.04.003 28408306

[47] Morgan CL, Nadas AS. Sweating and congestive heart failure. New England Journal of Medicine. 1963;268(11):580–5.

[48] WebMD Medical Reference from Healthwise. "Heart Failure: Less Common Symptoms—Topic Overview." WebMD. WebMD, n.d. Web. 28 June 2017.

[49] Iuliano S, Fisher SG, Karasik PE, Fletcher RD, Singh SN, Failure DoVASToATiCH. QRS duration and mortality in patients with congestive heart failure. American heart journal. 2002;143(6):1085–91. 12075267 10.1067/mhj.2002.122516

[50] Maheshwari S, Acharyya A, Puddu PE, Mazomenos EB, Leekha G, Maharatna K, Schiariti M. An automated algorithm for online detection of fragmented QRS and identification of its various morphologies. Journal of the Royal Society Interface. 2013;10(89):20130761. 10.1098/rsif.2013.0761 PMC3808554 24132202

[51] Naughton MT. Respiratory sleep disorders in patients with congestive heart failure. Journal of thoracic disease. 2015;7(8):1298 doi: 10.3978/j.issn.2072-1439.2015.07.02 26380758 10.3978/j.issn.2072-1439.2015.07.02 PMC4561277

[52] Cubbon RM, Ruff N, Groves D, Eleuteri A, Denby C, Kearney L, et al Ambulatory heart rate range predicts mode-specific mortality and hospitalisation in chronic heart failure. Heart. 2015: heartjnl-2015-308428. 10.1136/heartjnl-2015-308428 PMC4752612 26674986

[53] Greene SJ, Vaduganathan M, Wilcox JE, Harinstein ME, Maggioni AP, Subacius H, et al The prognostic significance of heart rate in patients hospitalized for heart failure with reduced ejection fraction in sinus rhythm: insights from the EVEREST (Efficacy of Vasopressin Antagonism in Heart Failure: Outcome Study With Tolvaptan) trial. JACC: Heart Failure. 2013;1(6):488–96. doi: 10.1016/j.jchf.2013.08.005 24622000 10.1016/j.jchf.2013.08.005

[54] Metra M. Tachycardia After a Heart Failure Hospitalization. JACC: Heart Failure; 2013. 10.1016/j.jchf.2013.10.003 24622001

