# Supplementary figures and images for: Mining telemonitored physiological data and patient-reported outcomes of congestive heart failure patients
Source: PLoS One. 2018 Mar 1;13(3):e0190323. doi: 10.1371/journal.pone.0190323 (PMC5832202; doi:10.1371/journal.pone.0190323)

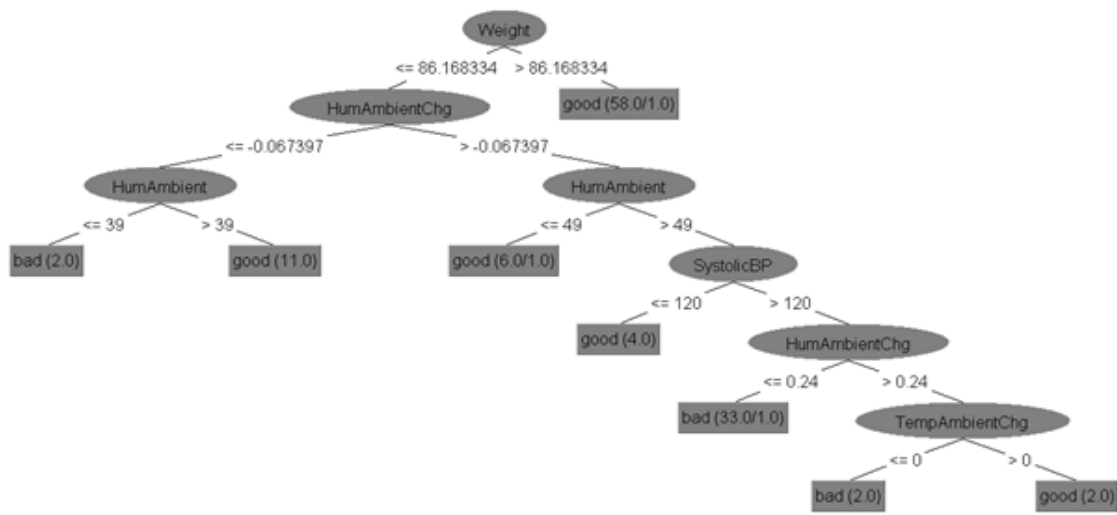

Supplement: S1 Fig — (PDF) [file pone.0190323.s001.pdf]

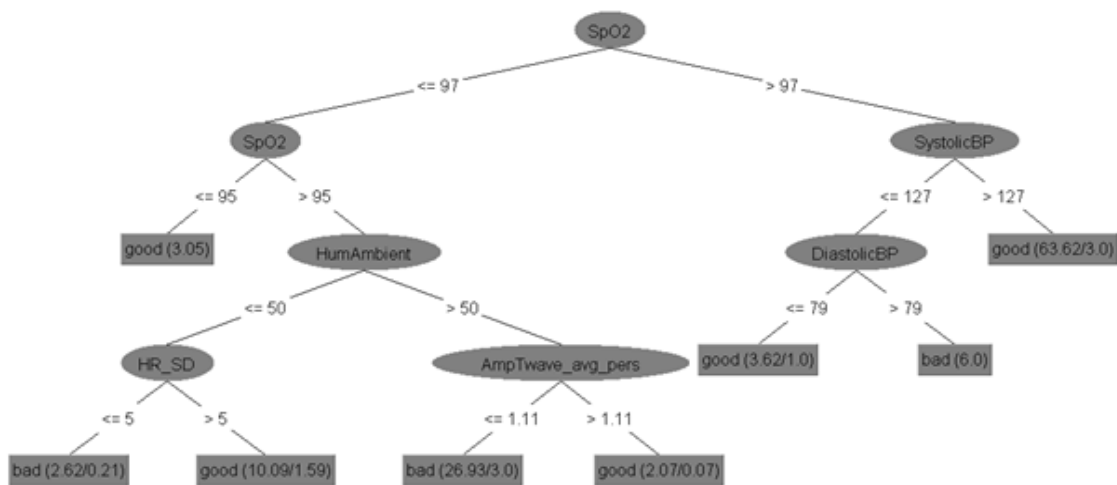

Supplement: S2 Fig — (PDF) [file pone.0190323.s002.pdf]

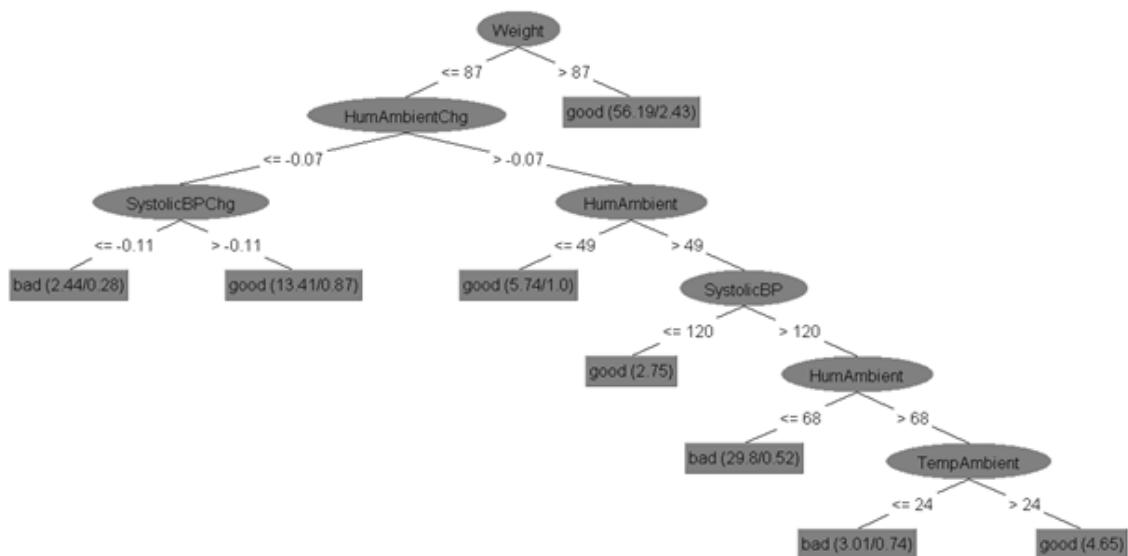

Supplement: S3 Fig — (PDF) [file pone.0190323.s003.pdf]

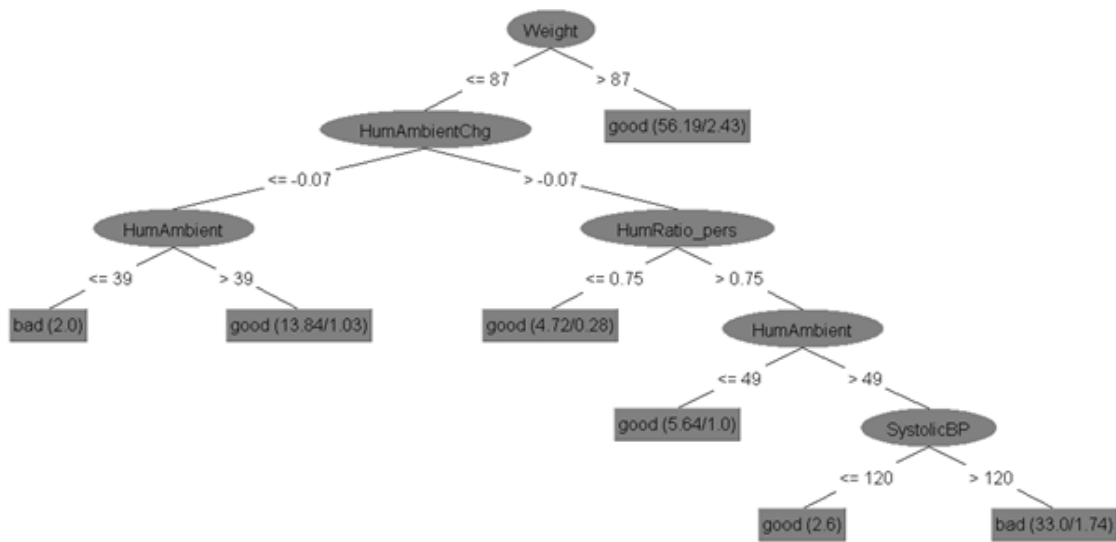

Supplement: S4 Fig — (PDF) [file pone.0190323.s004.pdf]

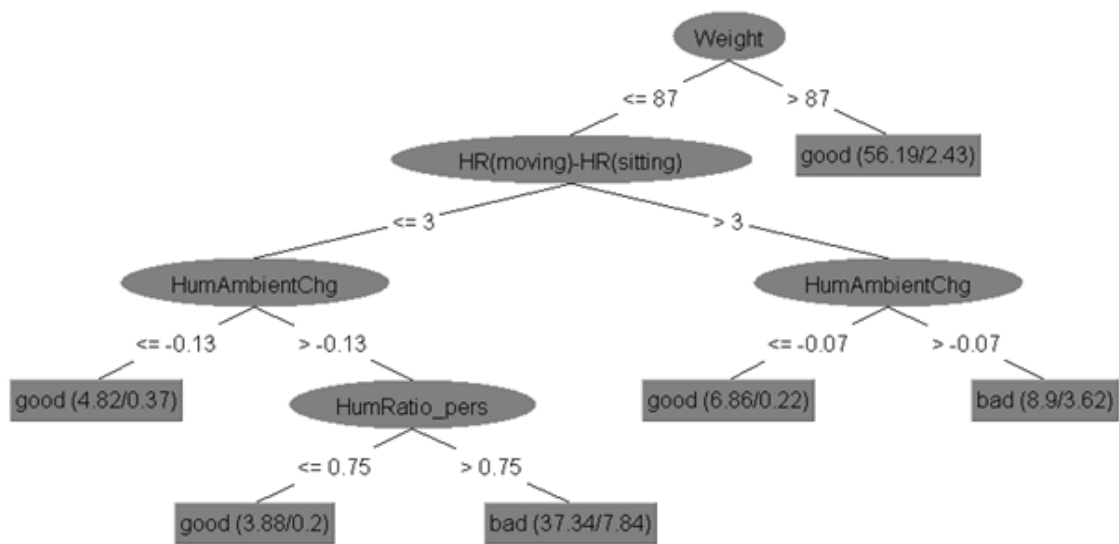

Supplement: S5 Fig — (PDF) [file pone.0190323.s005.pdf]

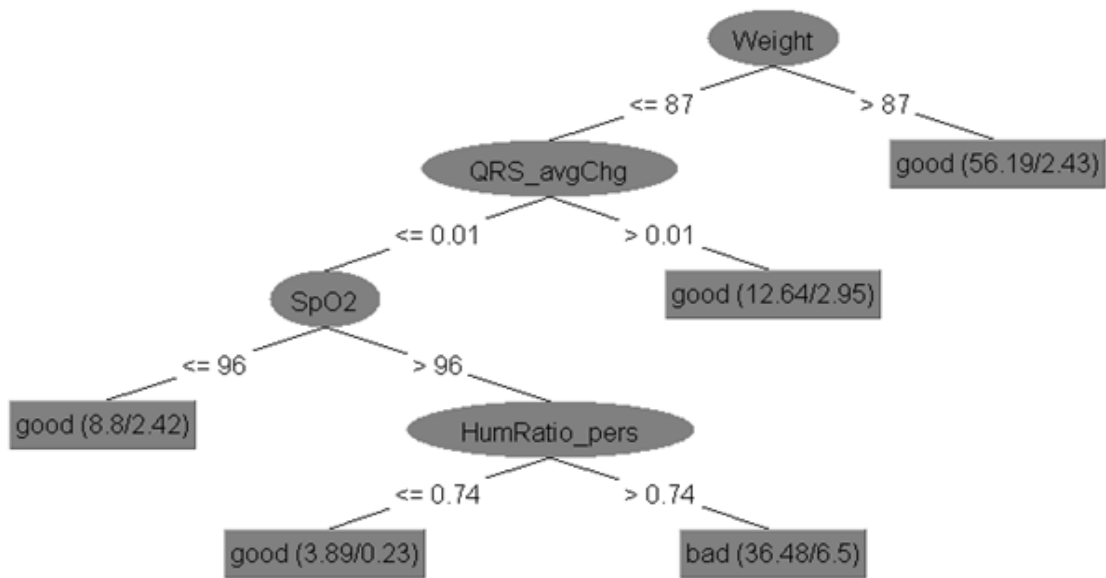

Supplement: S6 Fig — (PDF) [file pone.0190323.s006.pdf]

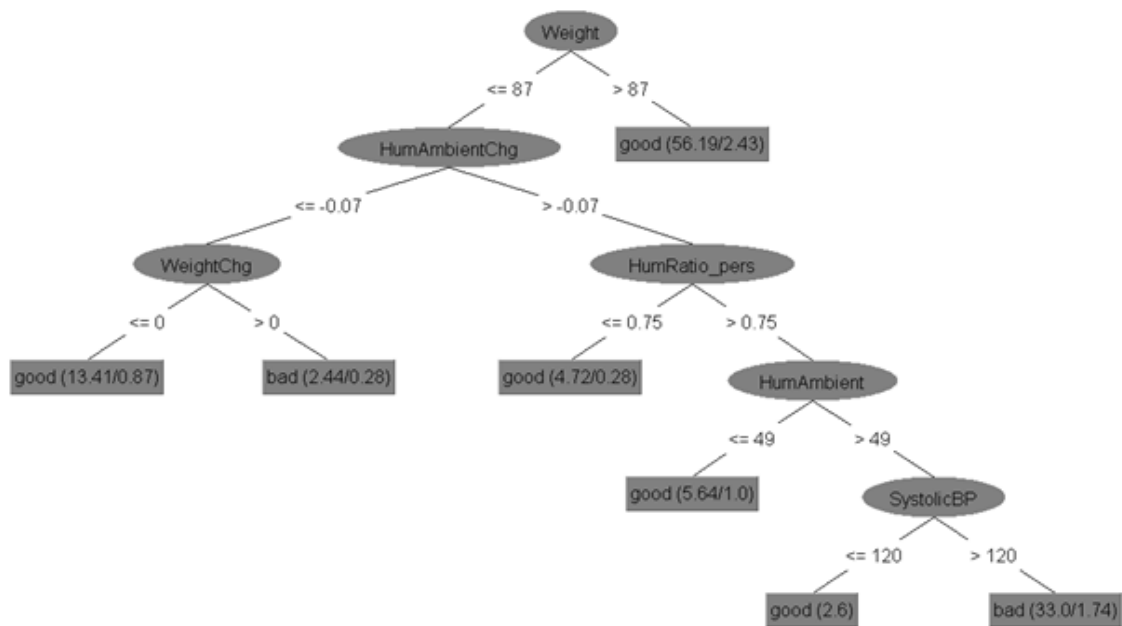

Supplement: S7 Fig — (PDF) [file pone.0190323.s007.pdf]

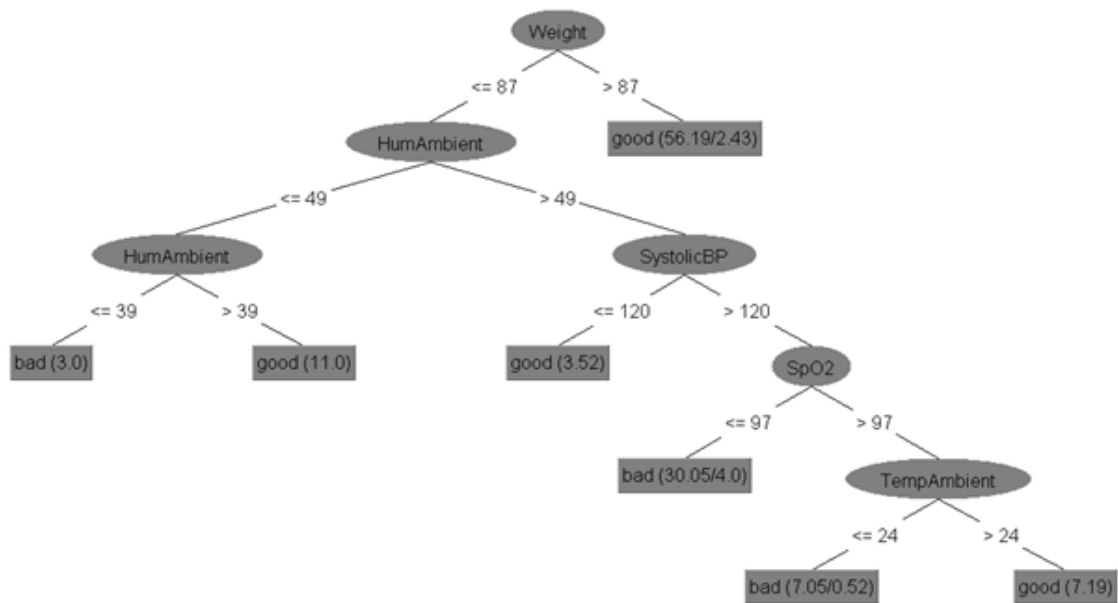

Supplement: S8 Fig — (PDF) [file pone.0190323.s008.pdf]

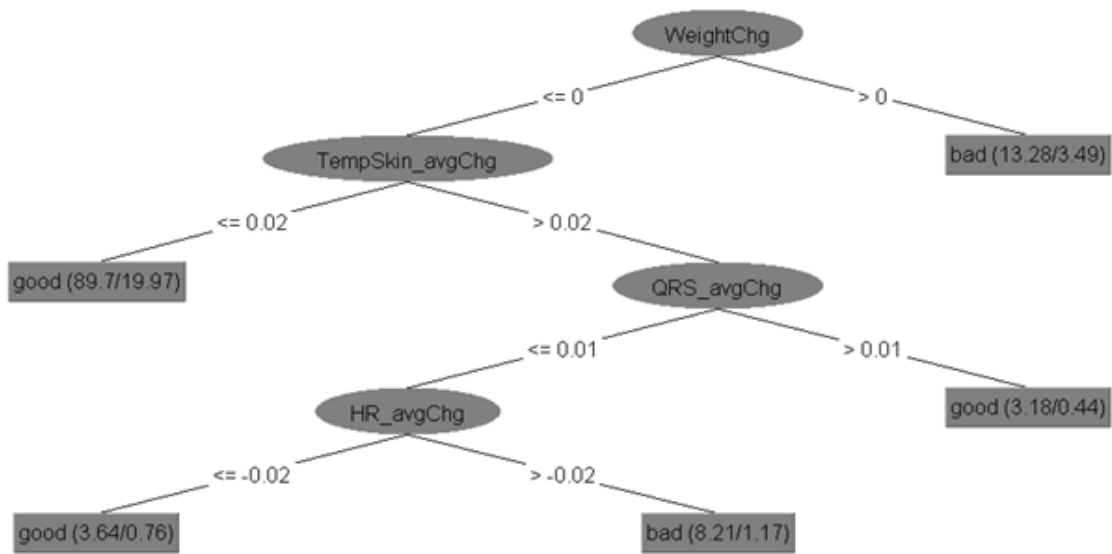

Supplement: S9 Fig — (PDF) [file pone.0190323.s009.pdf]

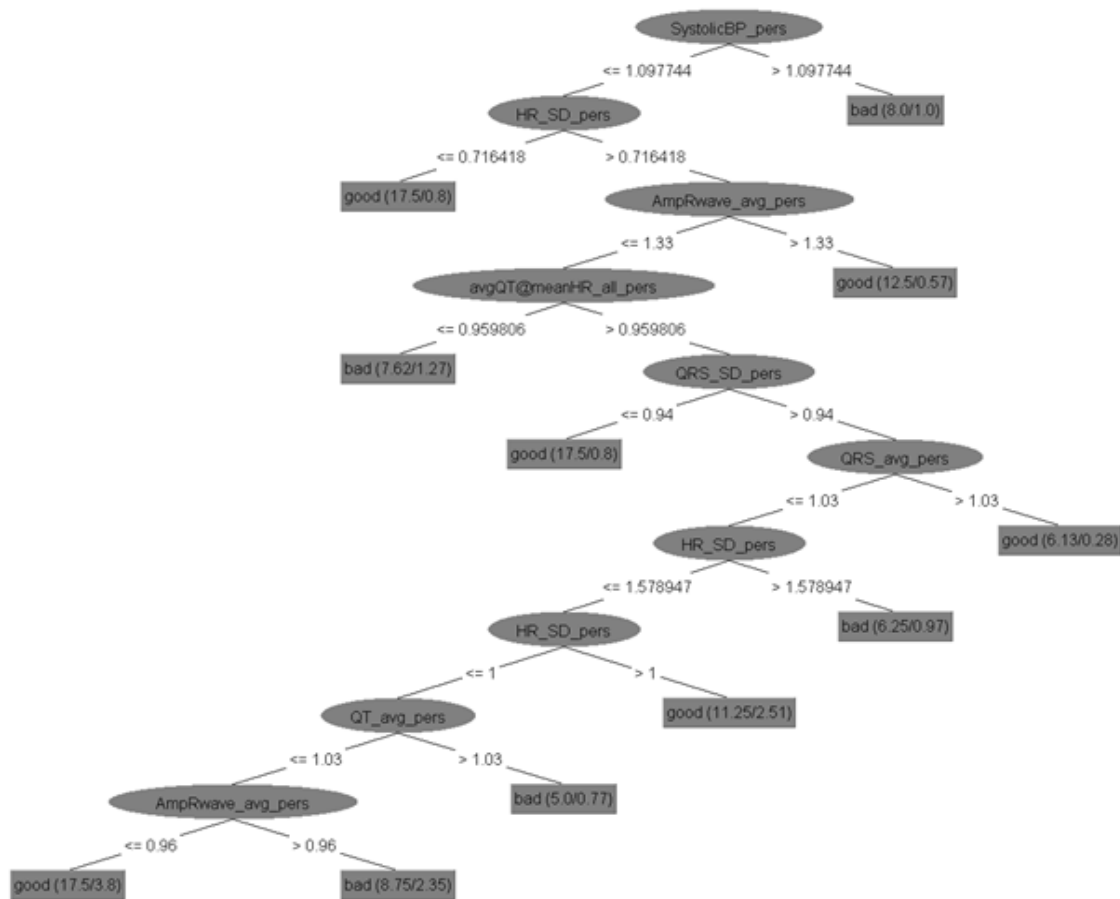

Supplement: S10 Fig — (PDF) [file pone.0190323.s010.pdf]
