# Supplementary material for: Mining telemonitored physiological data and patient-reported outcomes of congestive heart failure patients
Source: PLoS One. 2018 Mar 1;13(3):e0190323. doi: 10.1371/journal.pone.0190323 (PMC5832202; doi:10.1371/journal.pone.0190323)
Supplement: S1 Table — (DOCX) [file pone.0190323.s011.docx]

**S1 Table: The classification accuracy for each feature subsets and data mining algorithm averaged over all the class definitions.**

| **Algorithms**  **Subsets** | **Random forest** | **Decision tree** | **Naïve Bayes** | **SMO** | **Average all algorithms** | **Average RF and DT** | Majority |
| --- | --- | --- | --- | --- | --- | --- | --- |
| No_sparse_features_0.17_kNN: | 84.21 | 81.67 | 71.05 | 79.70 | **79.16** | **82.94** | 66.86 |
| No_sparse_features_0.17: | 83.11 | 78.46 | 69.84 | 80.17 | 77.90 | 80.79 | 66.86 |
| No_sparse_features_0.17_MICE: | 82.50 | 77.55 | 70.12 | 80.48 | 77.66 | 80.03 | 66.86 |
| Expert_selection: | 80.58 | 79.12 | 71.73 | 75.53 | 76.74 | 79.85 | 66.86 |
| CFS_feature_selection: | 80.32 | 78.96 | 74.98 | 78.12 | 78.09 | 79.64 | 66.86 |
| No_sparse_features_0.27: | 80.21 | 78.56 | 70.35 | 72.78 | 75.47 | 79.39 | 66.86 |
| No_sparse_features_0.17_SVD: | 81.36 | 76.67 | 70.71 | 80.15 | 77.22 | 79.01 | 66.86 |
| No_sparse_features_0.27_kNN: | 77.50 | 80.51 | 71.88 | 77.98 | 76.97 | 79.00 | 66.86 |
| CFS_feature_selection_kNN: | 79.52 | 77.06 | 74.13 | 77.69 | 77.10 | 78.29 | 66.86 |
| CFS_feature_selection_SVD: | 79.50 | 76.56 | 74.91 | 78.13 | 77.28 | 78.03 | 66.86 |
| Expert_selection_kNN: | 77.64 | 77.61 | 72.30 | 76.02 | 75.89 | 77.62 | 66.86 |
| No_sparse_features_0.27_SVD: | 75.41 | 79.61 | 71.41 | 74.49 | 75.23 | 77.51 | 66.86 |
| No_activities_avg_and_std_dev: | 79.65 | 75.36 | 72.20 | 70.96 | 74.54 | 77.51 | 66.86 |
| No_activities: | 77.34 | 77.18 | 68.37 | 66.57 | 72.36 | 77.26 | 66.86 |
| No_sparse_features_0.27_MICE: | 75.35 | 78.23 | 67.88 | 74.19 | 73.91 | 76.79 | 66.86 |
| No_activities_kNN: | 76.21 | 76.10 | 71.97 | 72.23 | 74.13 | 76.16 | 66.86 |
| Expert_selection_SVD: | 77.03 | 74.75 | 72.15 | 74.00 | 74.48 | 75.89 | 66.86 |
| CFS_feature_selection_MICE: | 75.69 | 75.16 | 74.55 | 77.18 | 75.64 | 75.42 | 66.86 |
| All: | 74.97 | 75.58 | 68.57 | 64.49 | 70.90 | 75.27 | 66.86 |
| Expert_selection_MICE: | 75.87 | 74.17 | 73.90 | 74.70 | 74.66 | 75.02 | 66.86 |
| No_activities_SVD: | 75.79 | 74.07 | 64.08 | 71.42 | 71.34 | 74.93 | 66.86 |
| No_activities_avg_and_std_dev_kNN: | 75.95 | 73.30 | 72.46 | 73.52 | 73.81 | 74.62 | 66.86 |
| All_SVD: | 76.05 | 72.63 | 72.28 | 65.95 | 71.73 | 74.34 | 66.86 |
| No_activities_avg_and_std_dev_SVD: | 76.09 | 72.48 | 66.75 | 73.49 | 72.20 | 74.28 | 66.86 |
| No_activities_MICE: | 74.13 | 73.78 | 70.75 | 69.22 | 71.97 | 73.95 | 66.86 |
| All_kNN: | 73.88 | 73.89 | 70.05 | 61.68 | 69.87 | 73.89 | 66.86 |
| No_activities_avg_and_std_dev_MICE: | 74.75 | 71.52 | 73.29 | 71.67 | 72.81 | 73.13 | 66.86 |
| All_MICE: | 70.63 | 71.67 | 72.18 | 70.22 | 71.17 | 71.15 | 66.86 |
| No_activities_personalised: | 72.04 | 67.72 | 61.01 | 55.94 | 64.18 | 69.88 | 66.86 |
| No_activities_changes: | 67.97 | 70.79 | 66.93 | 57.45 | 65.79 | 69.38 | 66.86 |
| No_activities_changes_kNN: | 70.66 | 67.28 | 62.22 | 56.65 | 64.20 | 68.97 | 66.86 |
| No_activities_changes_SVD: | 71.78 | 66.05 | 59.68 | 56.82 | 63.58 | 68.91 | 66.86 |
| No_activities_changes_MICE: | 70.58 | 66.20 | 65.57 | 52.63 | 63.75 | 68.39 | 66.86 |
| No_activities_personalised_MICE: | 69.08 | 64.56 | 59.50 | 58.21 | 62.84 | 66.82 | 66.86 |
| No_activities_personalised_kNN: | 69.07 | 64.55 | 59.99 | 61.95 | 63.89 | 66.81 | 66.86 |
| No_activities_personalised_SVD: | 69.03 | 63.45 | 58.63 | 58.37 | 62.37 | 66.24 | 66.86 |
| Average | **75.87** | 73.97 | 69.12 | 70.02 | 72.25 | 74.92 | 66.86 |
