# Supplementary material for: Mining telemonitored physiological data and patient-reported outcomes of congestive heart failure patients
Source: PLoS One. 2018 Mar 1;13(3):e0190323. doi: 10.1371/journal.pone.0190323 (PMC5832202; doi:10.1371/journal.pone.0190323)
Supplement: S2 Table — (DOCX) [file pone.0190323.s012.docx]

**S2 Table: The classification accuracy for each feature subset and data mining algorithms, averaged over all the class definitions, without imputation.**

| **Algorithms**  **Subsets** | **Random forest** | **Decision tree** | **Naïve Bayes** | **SMO** | **Average all algorithms** | **Average RF and DT** |
| --- | --- | --- | --- | --- | --- | --- |
| All | 74.97 | 75.58 | 68.57 | 64.49 | 70.90 | 75.27 |
| CFS_feature_selection | 80.32 | 78.96 | 74.98 | 78.12 | 78.09 | 79.64 |
| Expert_selection | 80.58 | 79.12 | 71.73 | 75.53 | 76.74 | 79.85 |
| No_activities | 77.34 | 77.18 | 68.37 | 66.57 | 72.36 | 77.26 |
| No_activities_avg_and_std_dev | 79.65 | 75.36 | 72.20 | 70.96 | 74.54 | 77.51 |
| No_activities_changes | 67.97 | 70.79 | 66.93 | 57.45 | 65.79 | 69.38 |
| No_activities_personalised | 72.04 | 67.72 | 61.01 | 55.94 | 64.18 | 69.88 |
| No_sparse_features_0.17 | 83.11 | 78.46 | 69.84 | 80.17 | **77.90** | **80.79** |
| No_sparse_features_0.27 | 80.21 | 78.56 | 70.35 | 72.78 | 75.47 | 79.39 |
| Average | **77.36** | 75.75 | 69.33 | 69.11 | 72.89 | **76.55** |
