# Supplementary material for: Mining telemonitored physiological data and patient-reported outcomes of congestive heart failure patients
Source: PLoS One. 2018 Mar 1;13(3):e0190323. doi: 10.1371/journal.pone.0190323 (PMC5832202; doi:10.1371/journal.pone.0190323)
Supplement: S3 Table — (DOCX) [file pone.0190323.s013.docx]

**S3 Table: The classification accuracy for each feature subset and data mining algorithms, averaged over all the class definitions, with kNN imputation**

| **Algorithms**  **Subsets** | **Random forest** | **Decision tree** | **Naïve Bayes** | **SMO** | **Average all algorithms** | **Average RF and DT** |
| --- | --- | --- | --- | --- | --- | --- |
| All: | 73.88 | 73.89 | 70.05 | 61.68 | 69.87 | 73.89 |
| CFS_feature_selection: | 79.52 | 77.06 | 74.13 | 77.69 | 77.10 | 78.29 |
| Expert_selection: | 77.64 | 77.61 | 72.30 | 76.02 | 75.89 | 77.62 |
| No_activities: | 75.95 | 73.30 | 72.46 | 73.52 | 73.81 | 74.62 |
| No_activities_avg_and_std_dev: | 70.66 | 67.28 | 62.22 | 56.65 | 64.20 | 68.97 |
| No_activities_changes: | 76.21 | 76.10 | 71.97 | 72.23 | 74.13 | 76.16 |
| No_activities_personalised: | 69.07 | 64.55 | 59.99 | 61.95 | 63.89 | 66.81 |
| No_sparse_features_0.17: | 84.21 | 81.67 | 71.05 | 79.70 | **79.16** | **82.94** |
| No_sparse_features_0.27: | 77.50 | 80.51 | 71.88 | 77.98 | 76.97 | 79.00 |
| Average | **76.07** | 74.66 | 69.56 | 70.82 | 72.78 | **75.37** |
