# Supplementary material for: Mining telemonitored physiological data and patient-reported outcomes of congestive heart failure patients
Source: PLoS One. 2018 Mar 1;13(3):e0190323. doi: 10.1371/journal.pone.0190323 (PMC5832202; doi:10.1371/journal.pone.0190323)
Supplement: S4 Table — (DOCX) [file pone.0190323.s014.docx]

**S4 Table: The classification accuracy for each feature subset and data mining algorithms, averaged over all the class definitions, with MICE imputation**

| **Algorithms**  **Subsets** | **Random forest** | **Decision tree** | **Naïve Bayes** | **SMO** | **Average all algorithms** | **Average RF and DT** |
| --- | --- | --- | --- | --- | --- | --- |
| All: | 70.63 | 71.67 | 72.18 | 70.22 | 71.17 | 71.15 |
| CFS_feature_selection: | 75.69 | 75.16 | 74.55 | 77.18 | 75.64 | 75.42 |
| Expert_selection: | 75.87 | 74.17 | 73.90 | 74.70 | 74.66 | 75.02 |
| No_activities: | 74.75 | 71.52 | 73.29 | 71.67 | 72.81 | 73.13 |
| No_activities_avg_and_std_dev: | 70.58 | 66.20 | 65.57 | 52.63 | 63.75 | 68.39 |
| No_activities_changes: | 74.13 | 73.78 | 70.75 | 69.22 | 71.97 | 73.95 |
| No_activities_personalised: | 69.08 | 64.56 | 59.50 | 58.21 | 62.84 | 66.82 |
| No_sparse_features_0.17: | 82.50 | 77.55 | 70.12 | 80.48 | **77.66** | **80.03** |
| No_sparse_features_0.27: | 75.35 | 78.23 | 67.88 | 74.19 | 73.91 | 76.79 |
| **Average** | **74.29** | 72.54 | 69.75 | 69.83 | 71.60 | **73.41** |
