# Supplementary material for: Mining telemonitored physiological data and patient-reported outcomes of congestive heart failure patients
Source: PLoS One. 2018 Mar 1;13(3):e0190323. doi: 10.1371/journal.pone.0190323 (PMC5832202; doi:10.1371/journal.pone.0190323)
Supplement: S5 Table — (DOCX) [file pone.0190323.s015.docx]

**S5 Table: The classification accuracy for each feature subset and data mining algorithms, averaged over all the class definitions, with SVD imputation.**

| **Algorithms**  **Subsets** | **Random forest** | **Decision tree** | **Naïve Bayes** | **SMO** | **Average all algorithms** | **Average RF and DT** |
| --- | --- | --- | --- | --- | --- | --- |
| All: | 76.05 | 72.63 | 72.28 | 65.95 | 71.73 | 74.34 |
| CFS_feature_selection: | 79.50 | 76.56 | 74.91 | 78.13 | 77.28 | 78.03 |
| Expert_selection: | 77.03 | 74.75 | 72.15 | 74.00 | 74.48 | 75.89 |
| No_activities: | 76.09 | 72.48 | 66.75 | 73.49 | 72.20 | 74.28 |
| No_activities_avg_and_std_dev: | 71.78 | 66.05 | 59.68 | 56.82 | 63.58 | 68.91 |
| No_activities_changes: | 69.03 | 63.45 | 58.63 | 58.37 | 62.37 | 66.24 |
| No_activities_personalised: | 75.79 | 74.07 | 64.08 | 71.42 | 71.34 | 74.93 |
| No_sparse_features_0.17: | 81.36 | 76.67 | 70.71 | 80.15 | **77.22** | **79.01** |
| No_sparse_features_0.27: | 75.41 | 79.61 | 71.41 | 74.49 | 75.23 | 77.51 |
| **Average** | **75.78** | 72.92 | 67.84 | 70.31 | 71.71 | **74.35** |
