# Supplementary material for: Mining telemonitored physiological data and patient-reported outcomes of congestive heart failure patients
Source: PLoS One. 2018 Mar 1;13(3):e0190323. doi: 10.1371/journal.pone.0190323 (PMC5832202; doi:10.1371/journal.pone.0190323)
Supplement: S6 Table — The references in table are provided only for relation types not discussed in the body of the paper. (DOCX) [file pone.0190323.s016.docx]

**S6 Table: Relations obtained from the ten decision trees in Figs. S1-S10 with their types and cut-off values. The references are provided only for relation types not discussed in the body of the paper.**

| **Relation** | **Type** | **DS1** | **DS2** | **DS3** | **DS4** | **DS5** | **DS6** | **DS7** | **DS8** | **DS9** | **DS10** |
| --- | --- | --- | --- | --- | --- | --- | --- | --- | --- | --- | --- |
| AmpRwave_avg_pers large: good | Against [1,2] |  |  |  |  |  |  |  |  |  | 1.33 |
| AmpRwave_avg_pers small: good, large: bad | In favor [3] |  |  |  |  |  |  |  |  |  | 0.96 |
| AmpTwave_avg_pers small: bad, large: good |  |  | 1.11 |  |  |  |  |  |  |  |  |
| DiastolicBP large: bad (SystolicBP small) | In favor [4] |  | 79 |  |  |  |  |  |  |  |  |
| DiastolicBP small: good |  |  | 79 |  |  |  |  |  |  |  |  |
| SystolicBP small: bad, large: good | In favor [4] |  | 127 |  |  |  |  |  |  |  |  |
| SystolicBP small: good, large: bad | Against [4] | 120 |  | 120 | 120 |  |  | 120 | 120 |  |  |
| SystolicBP_pers large: bad | Against [4] |  |  |  |  |  |  |  |  |  | 1.1 |
| SystolicBPChg small: bad, large: good | In favor [4] |  |  | -0.11 |  |  |  |  |  |  |  |
| HR(moving)-HR(sitting) small: bad | In favor [5] |  |  |  |  | 3 |  |  |  |  |  |
| HR_avgChg small: good, large: bad | In favor [5] |  |  |  |  |  |  |  |  | -0.02 |  |
| HR_SD small: bad, large: good | In favor [5] |  | 5 |  |  |  |  |  |  |  |  |
| HR_SD_pers large: good | In favor [5] |  |  |  |  |  |  |  |  |  | 1 |
| HR_SD_pers small: good, large: bad | Against [5] |  |  |  |  |  |  |  |  |  | 1.58 |
| HumAmbient small: bad | Against [6] |  |  | 68 |  |  |  |  |  |  |  |
| HumAmbient small: good, large: bad |  | 49 | 50 | 49 | 49 |  |  | 49 | 49 |  |  |
| HumAmbient very small: bad, large: good |  | 39 |  |  | 39 |  |  |  | 39 |  |  |
| HumAmbientChg small: bad, large: good | Against [6] | 0.24 |  |  |  |  |  |  |  |  |  |
| HumAmbientChg small: good, large: bad |  | -0.07 |  | -0.07 | -0.07 | -0.13, -0.07 |  | -0.07 |  |  |  |
| HumRatio small: good, large: bad |  |  |  |  | 0.75 |  |  |  |  |  |  |
| HumRatio_pers small: good, large: bad |  |  |  |  |  | 0.75 | 0.74 | 0.75 |  |  |  |
| QRS_avg_pers large: good | Against [7] |  |  |  |  |  |  |  |  |  | 1.03 |
| QRS_avgChg large: good, small: bad | Against [7] |  |  |  |  |  | 0.01 |  |  | 0.01 |  |
| QRS_SD_pers small: good |  |  |  |  |  |  |  |  |  |  | 0.94 |
| QT_avg_pers large: bad | In favor [8] |  |  |  |  |  |  |  |  |  | 1.03 |
| AvgQT@meanHR_all_pers small: bad | Against [8] |  |  |  |  |  |  |  |  |  | 0.96 |
| SpO2 small: bad, large: good | In favor [9] |  | 97 |  |  |  |  |  | 97 |  |  |
| SpO2 small: good, large: bad | Against [9] |  | 95 |  |  |  | 96 |  |  |  |  |
| TempAmbient small: bad, large: good (HumAmbient large) |  |  |  | 24 |  |  |  |  | 24 |  |  |
| TempAmbientChg small: bad, large: good |  | 0 |  |  |  |  |  |  |  |  |  |
| TempSkin_avgChg small: good, large: bad |  |  |  |  |  |  |  |  |  | 0.02 |  |
| Weight large: good, small: bad | Against [10] | 86.2 |  | 87 | 87 | 87 | 87 | 87 | 87 |  |  |
| WeightChg small: good, large: bad | In favor [10] |  |  |  |  |  |  | 0 |  | 0 |  |

# References

1. Hakki, A-Hamid, et al. "R wave amplitude: a new determinant of failure of patients with coronary heart disease to manifest ST segment depression during exercise." Journal of the American College of Cardiology 3.5 (1984): 1155-1160.
2. Ohlmeier, H., H. Mannebach, and U. Gleichmann. "Correlation between R‐wave amplitude and left ventricular volume at rest and with exercise." Clinical cardiology 6.1 (1983): 29-36.
3. Bonoris, Peter E., et al. "Evaluation of R wave amplitude changes versus ST-segment depression in stress testing." Circulation 57.5 (1978): 904-910.
4. Lee, Tobias T., et al. "The association between blood pressure and mortality in patients with heart failure." American heart journal 151.1 (2006): 76-83.
5. Cubbon, Richard M., et al. "Ambulatory heart rate range predicts mode-specific mortality and hospitalisation in chronic heart failure." Heart (2015): heartjnl-2015.
6. Goggins, William B., and Emily YY Chan. "A study of the short-term associations between hospital admissions and mortality from heart failure and meteorological variables in Hong Kong: Weather and heart failure in Hong Kong." International Journal of Cardiology 228 (2017): 537-542.
7. Iuliano, Stephen, et al. "QRS duration and mortality in patients with congestive heart failure." American heart journal 143.6 (2002): 1085-1091.
8. "What Is Long QT Syndrome?" National Heart Lung and Blood Institute. U.S. Department of Health and Human Services, 21 Sept. 2011. Web. 30 June 2017.
9. Naughton, Matthew T. "Respiratory sleep disorders in patients with congestive heart failure." Journal of thoracic disease 7.8 (2015): 1298-1310.
10. Puddu, Paolo Emilio, et al. "A clinical observational study in the CHIRON project: rationale and expected results." International Conference on Smart Homes and Health Telematics. Springer Berlin Heidelberg, 2012.
